# Comparison of Immune Checkpoint Inhibitors between Older and Younger Patients with Advanced or Metastatic Lung Cancer: A Systematic Review and Meta-Analysis

**DOI:** 10.1155/2019/9853701

**Published:** 2019-05-13

**Authors:** Leyin Zhang, Leitao Sun, Jieru Yu, Feiyu Shan, Kai Zhang, Xi Pang, Chenghao Ma, Yinan Zhang, Minhe Shen, Shenglin Ma, Shanming Ruan

**Affiliations:** ^1^The First Clinical Medical College of Zhejiang Chinese Medical University, Hangzhou 310053, Zhejiang, China; ^2^College of Basic Medical Science, Zhejiang Chinese Medical University, Hangzhou 310053, Zhejiang, China; ^3^The First Affiliated Hospital of Wenzhou Medical University, Wenzhou 325000, Zhejiang, China; ^4^Department of Medical Oncology, The First Affiliated Hospital of Zhejiang Chinese Medical University, Hangzhou 310006, Zhejiang, China; ^5^Department of Radiotherapy, The Fourth Clinical Medical College of Zhejiang Chinese Medical University, Hangzhou 310006, Zhejiang, China

## Abstract

**Objectives:**

Despite the fact that it is widely acknowledged that immune checkpoint inhibitors (ICIs) rely on the presence of immune response to take their antitumor effect, little is known whether there is an influence exerted on the efficacy of ICIs based on patients' age. We performed a systematic review and meta-analysis to explore the efficacy of ICIs between younger and older patients.

**Materials and Methods:**

We searched online database and major conference proceedings for randomized controlled trials (RCTs) published of ICIs and included RCTs that conducted subgroup comparisons of age with available combination of hazard ratios (HRs) and 95% confidence interval (95%CI). Subsequently, we figured out the pooled HR and 95%CI in younger and older patients with a random-effects model and evaluated the within-study heterogeneity by using subgroup, sensitivity, and meta-regression analysis.

**Results and Conclusion:**

A total of 12 eligible RCTs included in our study, which reported OS according to patients' age. The overall estimated random-effects for HR was 0.75 with 95% CI of 0.65-0.87 in younger arm versus 0.81 with 95% CI of 0.72-0.92 in older arm. ICIs can improve OS for patients with advanced or metastatic lung cancer when compared to controls, especially for those patients with NSCLC, anti-PD-1/PD-L1 inhibitors, non-squamous, Pembrolizumab or Atezolizumab used as well as subsequent-line setting, and the magnitude of benefit in OS had comparable efficacy in both younger and older arms using a cut-off of 65 yr. Conversely, we also drew a statically significant conclusion that older patients failed to acquire benefit from ICIs when subdivided with a further cut-off of 75 yr.

## 1. Introduction

Lung cancer, one of the most common malignant tumors, is currently the predominant cause of cancer-related deaths worldwide, which is responsible for more than 1.80 million deaths annually [1], and the 5-year survival rate has still remained poor at 16% [2]. Over half of patients with lung cancer have locally advanced or systemic metastasis leading to losing the opportunity for surgical resection. Thus, platinum-based doublet chemotherapy and radiotherapy are often regarded as the primary choice for patients [3]. However, even with these diversified therapies [4], the majority of lung patients will not acquire a satisfied prognosis. Hence, in recent years, the research on the antitumor activity of the immune system has resulted in the study and application of immunotherapy as the leading focus in lung cancer [5].

Age is a well-known risk factor for development and progression of cancer [6] and is also associated with a poorer prognosis [7, 8]. Likewise, it has been embroiled in debate that if older patients may benefit less from immune checkpoint inhibitors (ICIs), age-associated alterations to the immune system may lead to the reduction of immune function [9, 10]. In xenograft, mouse models have verified that age is connected with changes in immune system [11, 12]. When compared with younger ones, older mice further proved modificative cytokine kinetics [13] and reduced CD8^+^T cell proliferation [14], and this is also associated with the decrement in T cell function [15] and CD28 expression [16, 17], which is a co-stimulatory signal for T cell activation [18]. But in clinical trials, Elias et al. [19] reviewed efficacy and safety of ICI in patients with non-small cell lung cancer, melanoma, and renal cancer and found that there were no obvious age-associated difference in overall survival (OS) and side effects among those older and younger patients. Interestingly, in CheckMate 066, which regarded Nivolumab as a first-line treatment in melanoma, showed a more favorable and significant hazard ratio (HR) for disease progression or cancer death in patients older than 75 yr [20]. As for CheckMate 069, the objective response rate was about 64% in patients <65 yr compared to 53% in those aged 65 or older [21].

While the data are conflicting, the clinical efficacy of immunotherapy has not been fully elucidated in previous clinical trials. We did a systematic review and meta-analysis to assess if there is age-dependent influence in patients with advanced or metastatic lung cancer treated on ICI.

## 2. Methods

### 2.1. Data Source

We searched PubMed, Embase, and Cochrane for the most update of phase II and III randomized controlled trials (ICIs) published from the inception of each database to Oct 1, 2018. We only included the most complete and recent trial when duplicate publications were identified. Two investigators (L.Z. and L.S.) independently retrieved all the related studies in the databases and excluded duplicate publications. The combined text and medical subject heading (MeSH) terms were cross-searched using MeSH and free word as follows: (Immunotherapy [MeSH Terms] OR immunother*∗* [Title/Abstract] OR CTLA-4 [Title/Abstract] OR cytotoxic T-lymphocyte-associated protein 4 [Title/Abstract] OR PD-1 [Title/Abstract] OR programmed death receptor 1 [Title/Abstract] OR programmed death ligand1 [Title/Abstract] OR PD-L1 [Title/Abstract] OR immune checkpoint inhibitor [Title/Abstract] OR Ipilimumab [Title/Abstract] OR Tremelimumab [Title/Abstract] OR Nivolumab [Title/Abstract] OR Pembrolizumab [Title/Abstract] OR Durvalumab [Title/Abstract]) AND (lung neoplasms [MeSH Terms] OR (lung [Title/Abstract]) OR pulmonary [Title/Abstract] OR bronchus [Title/Abstract] OR bronchogenic [Title/Abstract] OR bronchial [Title/Abstract] OR bronchoalveolar [Title/Abstract] OR alveolar [Title/Abstract]) AND (cancer [Title/Abstract] OR carcinoma*∗* [Title/Abstract] OR neoplasm*∗* [Title/Abstract]) OR malignan*∗* [Title/Abstract] OR tumor [Title/Abstract])) AND (randomized controlled trial [Publication Type] OR controlled clinical trial [Publication Type] OR randomized [Title/Abstract] OR placebo [Title/Abstract] OR drug therapy [MeSH Subheading] OR randomly [Title/Abstract] OR trial [Title/Abstract] OR groups [Title/Abstract] NOT (animals [MeSH Terms] NOT (humans [MeSH Terms]) AND animals [MeSH Terms]). We also reviewed abstracts and presentations from major conference proceedings up to Oct 1, 2018 to ensure that no additional studies were overlooked.

### 2.2. Selection Criteria

Our meta-analysis is reported in line with the Preferred Reporting Items for Systematic Reviews and Meta-Analyses (PRISMA) Statement and had been registered at International Prospective Register of Systematic Reviews (number: CRD42018109933) [22]. RCTs meeting all of the following criteria were included: (1) Randomized controlled phase II or III studies in patients who were diagnosed with advanced or metastatic lung cancer; (2) ICIs includes PD-1, CTLA-4 or their combination; (3) In intervention group, ICIs were administered alone or in combination with other drugs, such as chemotherapy or other immunological drugs; (4) Participants are treated with control regimen without an ICIs; (4) Studies have data available for HR and 95% credible interval (CI) of OS based on a cut-off age.

Two independent investigators (L.Z. and L.S.) screened each reference by their titles and abstracts to elect potentially relevant articles meeting the predefined inclusion criteria, then looked through the full text of relevant articles from first selection. All disagreements about selection between investigators were discussed and resolved by all investigators.

### 2.3. Risk of Methodological Bias Assessment

Two independent investigators (L.Z. and L.S.) subjectively evaluated the quality of all studies according to the Cochrane evaluation handbook of RCTs (5.1.0), which includes random sequence generation, allocation concealment, blinding of participants, personnel and outcome assessment, incomplete outcome data, selective reporting and other bias, and then categorized it into three levels of “Yes” for a low risk of bias, “No” for a high risk of bias and “Unclear” for excepting the two situations mentioned above.

### 2.4. Data Extraction

Three investigators (X.P., C.M., and Y.Z.) independently performed data extraction and recording in a standard form. The following information was acquired from each included study: (1) Study characteristics: first author, publication time, study design, follow-up, phase and treatment arms; (2) Study population: number in each arm, median age, age range; (3) Study outcomes: HR and 95% CI for OS based on age subgroup. In case of trials that did not include survival subgroup analysis by age, we also reviewed each clinical trial's supplement.

### 2.5. Statistical Analysis

The individual data was extracted from each included trial on the basis of the steps mentioned in previous [23]. Three investigators (L.Z., L.S., and J.Y.) performed statistical analysis using STATA 15 and Review Manager (RevMan) 5.3. All data were expressed as the combination of HR and 95% CI, and* P<0.05* was considered to be statistically significant. Then STATA 15 was used to pool the data and produced the forest plots. We assessed the between-study heterogeneity by using the I^2^ test, which estimates the percentage of total variability across all studies. If the test showed *I* ^2^ *>50%* or* P<0.10*, the data were calculated through a random‐effects model. In addition, I^2^ regarded an estimated value that applied three fixed knots at 25%, 50%, and 75% as an indicator of mild, moderate, and high heterogeneity [24]. Otherwise, a fixed‐effects model was used to pool effect size. To deeply explore the heterogeneity and its potential influence, subgroup analysis was performed according to histotype, pathological type, type of ICI and line of treatment. Beyond that, meta-regression analysis was employed to examine which other characteristics might be the possible source of heterogeneity.

A funnel plot was used to estimate the potential publication bias when including studies reaching to ten or more. In addition to this, publication bias was also estimated by Egger's test and Begg's test, and* P<0.05 *indicated statistical significance. Sensitivity analysis, which examined the robustness of included trials to different aspects from methodological bias, was performed by step-wise removal of single study.

## 3. Results

### 3.1. Identification and Selection

**Figure 1:**
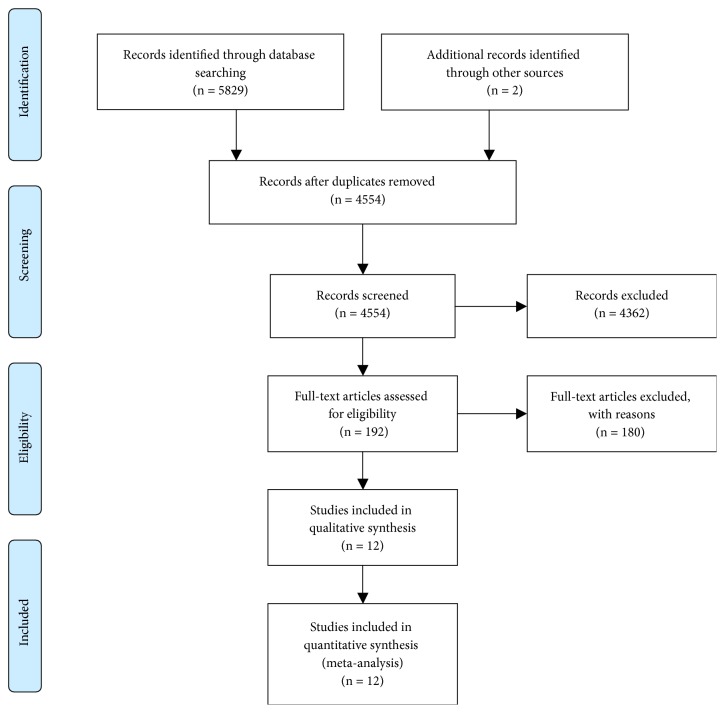
Articles retrieved and assessed for eligibility. After screening process, 12 RCT articles met the including criteria and were included in ultimate analysis.

After removal of 1277 duplicate articles from online database and other sources, a total of 4554 citations were left for preliminary screening, from which we selected 192 potentially relevant publications that match our inclusion criteria. And 4362 articles were excluded for one of the following reasons: Not RCTs, Not about advanced or metastatic lung cancer, Not with ICIs, Conference reports, Systematic reviews and Meta-analysis, Case report, Abstract articles review, With ICIs in control group, Single arm study. After full-text assessment, 180 articles were excluded as they did not contain the combination of HR and 95%CI calculated from OS subgroup analysis by age. In the end, 12 RCTs (Brahmer 2015 [25], Borghaei 2015 [26], Herbst 2015 [27], Reck 2016 [28], Carbone 2017 [29], Govindan 2017 [30], Gandhi 2018 [31], Paz-Ares 2018 [32], Horn 2018 [33], Antonia 2018 [34], Barlesi 2018 [35], Fehrenbacher 2018 [36]) were left for further analysis. The specific search and selection steps are shown in Figure 1.

### 3.2. Characteristics of Included Studies and Patients

There were a total of 8176 patients enrolled for the analysis of HRs for OS, 5475 males (67%) and 2702 females (33%). The age of participants ranged from 21 to 90 across all studies, and 3730 (46%) patients were older than 65 yr. The median follow-up ranged from 10.2 to 26.0 months. The main characteristics and results in each trial are presented in Table 1, and Supplementary Table 1.

### 3.3. Assessment of Methodological Bias

The random sequence was generated by using an interactive voice or web response system in only six trials (Barlesi 2018; Horn 2018; Paz-Ares 2018; Gandhi 2018; Herbst 2015; Reck 2016). Except for two trials (Herbst 2015; Barlesi 2018), other trials did not provide the detailed information about the allocation concealment. All trials provided the detailed information about the blinding of the participants and personnel and except one trial (Fehrebacher 2018), the remaining trials have low risk detection bias. Selective reporting existed in one trial (Reck 2016) and failed to completely report the end points originally decided. Except for five trials (Antonia 2018; Borghaei 2015; Carbone 2017; Paz-Ares 2018; Reck 2016), other trials had no obvious other bias. The assessment of risk of reporting bias, attrition bias, and other bias within each individual trials is listed in the supplement (Supplementary Figures 1 and 2).

### 3.4. Survival According to Age

**Figure 2:**
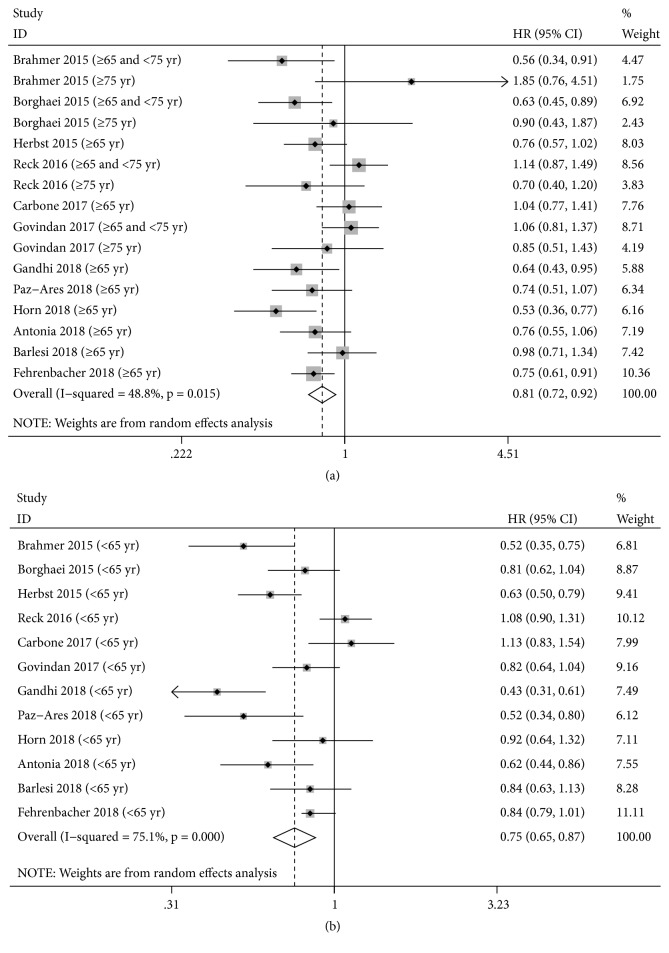
Forest plot of the meta-analysis estimating the hazard ratios and 95%CI of overall survival for older (a) and younger (b) patients assigned to intervention treatment, compared with those assigned to control treatment, by age. Squares represent study-specific HRs, and the size of square represents the weight of individual study included in the meta-analysis. Horizontal lines crossing the square indicate the 95% CIs. The dashed vertical lines indicate the age-specific pooled HR. Diamonds indicate the estimated overall effect according to meta-analysis random effect of pooled HRs from all included studies, calculated separately in younger and older patients, with their corresponding 95%CIs. The p value for heterogeneity is from the meta-analysis of the interaction HRs and represents heterogeneity by patients' age.

Compared with patients in control groups, the pooled HR was 0.81 with 95% CI of 0.72 to 0.92 in older arm treated with ICIs (Figure 2(a)). For younger patients, ICIs also significantly improved OS slightly higher than that in older arm (HR, 0.75; 95% CI, 0.65-0.87; Figure 2(b)), which showed that younger patients receive a comparable OS benefit from ICIs in comparison with older patients. Based on the included trials, there was substantially high heterogeneity among within-study in younger arm (I^2^=75.1%,* P≤0.001*), but mild heterogeneity in older arm (I^2^=48.8%,* P=0.015*), suggesting that the pooled estimate was supposed to be calculated based on the random-effects model. What is more, after subdividing the older arm with 75 yr as a further cut-off, there exists no obvious statistically significance in the two subsets (≥65 and <75 yr: HR: 0.84, 95% CI: 0.60-1.17,* P=0.298*; ≥75 yr: HR: 0.90, 95% CI: 0.64-1.25,* P=0.520*), and just mild heterogeneity was observed in ≥75 yr subset (I^2^=10.7%,* P=0.339*) (Supplementary Figure 3).

### 3.5. Subgroup Analysis

We explored within-study heterogeneity in the subgroup analysis based on type of ICIs, class of ICIs, pathogenic type, cancer histotype, line of treatment, and blind method given in the intervention group. The specific subgroup analysis outcomes are shown in Tables 2(a) and 2(b), and Supplementary Figures 4-9.

We found an advantage in favor of anti-PD-1/PD-L1 inhibitors in both arms with statistically significance, while no such difference was detected in anti-CTLA-4 inhibitors. Besides, the efficacy of anti-PD-1/PD-L1 inhibitors was slightly higher for younger patients (HR: 0.71, 95% CI: 0.60 to 0.84) than for older patients (HR: 0.76, 95% CI: 0.67 to 0.87). Additionally, the heterogeneity for age-related interaction in older arm, which was assessed between two subgroups, was still mild in anti-PD-1/PD-L1 inhibitors (I^2^ = 37.1%,* P=0.094*), but nearly none anti-CTLA-4 inhibitors (I^2^ = 0.7%,* P=0.388*).

For non-squamous subset, difference was only statistically significant in older arm, suggesting cancer histotype is considerable variability among patients aged 65 yr or older. And the analysis demonstrated that the risk for OS was not associated with histotype in younger arm (HR: 0.60; 95% CI, 0.32 to 1.11). And older patients showed a less heterogeneous cohort (I^2^ = 0.0%,* P=0.678*) when compared to younger patients (I^2^ = 88.2%,* P=0.004*). But for squamous subset, no statistical difference was demonstrated in terms of OS (HR: 0.87, 95% CI: 0.65 to 1.17) in older arm while receiving a better OS benefit in younger arm (HR: 0.63, 95% CI: 0.45 to 0.88).

Subgroup analysis also showed that there was no significant difference in OS when ICIs were used as first-line treatment both for younger patients (HR: 0.89, 95% CI: 0.71 to 1.12) and older patients (HR: 0.87, 95% CI: 0.71 to 1.07). Moreover, it revealed that younger patients who received subsequent-line treatment of ICIs obtained a comparable OS benefit (HR: 0.67, 95% CI: 0.56 to 0.81) in comparison with older patients (HR: 0.76, 95% CI: 0.66 to 0.86).

To reveal the influence of blind methods, subgroup analysis showed that both open-label and double-blind could come to an outcome with a statistical significance. When taking open-label into consideration, that offers roughly the same outcomes between older patients (HR: 0.81, 95% CI: 0.69 to 0.97) and younger patients (HR: 0.78, 95% CI: 0.66 to 0.93) for whom HRs were almost similar. But for double-blind, subgroup analysis indicated that the benefit from ICIs appeared to be comparable between younger patients (HR: 0.71, 95% CI: 0.53 to 0.95) and older patients (HR: 0.80, 95% CI: 0.66 to 0.98) when considered separately.

Furthermore, there was an evident trend to favor ICIs than control therapies in cancer patients with Pembrolizumab (younger: HR: 0.54, 95% CI: 0.42 to 0.68; older: HR: 0.72, 95% CI: 0.59 to 0.88) and Atezolizumab (younger: HR: 0.85, 95% CI: 0.75 to 0.95; older: HR: 0.66, 95% CI: 0.47 to 0.91) both in younger and older arm with statistically significance, but disfavor those in cancer patients who take Nivolumab (younger: HR: 0.79, 95% CI: 0.53 to 1.18; older: HR: 0.83, 95% CI: 0.59 to 1.18) and Ipilimumab (younger: HR: 0.95, 95% CI: 0.73 to 1.25; older: HR: 1.02, 95% CI: 0.86 to 1.21). Subgroup analysis reduced the high heterogeneity to mild of Pembrolizumab (I^2^ = 42.0%,* P=0.178*), to none of Atezolizumab (I^2^ = 0.0%,* P=0.641*), and to moderate of Ipilimumab (I^2^ = 67.7%,* P=0.079*) in younger arm, when older patients showed a less heterogeneity in Pembrolizumab (I^2^ = 0.0%,* P=0.782*), Atezolizumab (I^2^ = 60.2%,* P=0.113*), and Ipilimumab (I^2^ = 0.7%,* P=0.388*).

Finally, we performed a subgroup analysis according to pathology in SCLC versus NSCLC. For NSCLC, younger patients obtained a comparable benefit than older patients, yielding a HR of 0.71 with 95%CI of 0.60 to 0.83. But for SCLC, the HR of OS was not associated with age-related factor whether in younger arm (HR: 1.04, 95% CI: 0.88 to 1.23) or older arm (HR: 0.76, 95% CI: 0.45 to 1.29) with statistical significance. Separately, the combination of ICIs and standard chemotherapy in SCLC did not show a tendency to improve OS for younger and older patients. Beyond that, after carrying out a pathology subgroup analysis in younger arm, it revealed substantial heterogeneous decrease from high to none (I^2^ = 0.0%,* P=0.441*) in SCLC, and from high to moderate (I^2^ = 71.6%,* P≤0.001*) in NSCLC.

### 3.6. Meta-Regression Analysis

We performed the univariate meta-regression analysis to assess the correlation of different variables in patients treated with ICIs on the basis of age factor, after that the outcome of meta-regression demonstrated a statistically significant relationship for masking method (*P=0.026*) in younger arm and histotype (*P=0.010*), masking method (*P=0.039*) in older arm as a function of influencing heterogeneity, as shown in Supplementary Figures 10 and 11.

### 3.7. Publication Bias

**Figure 3:**
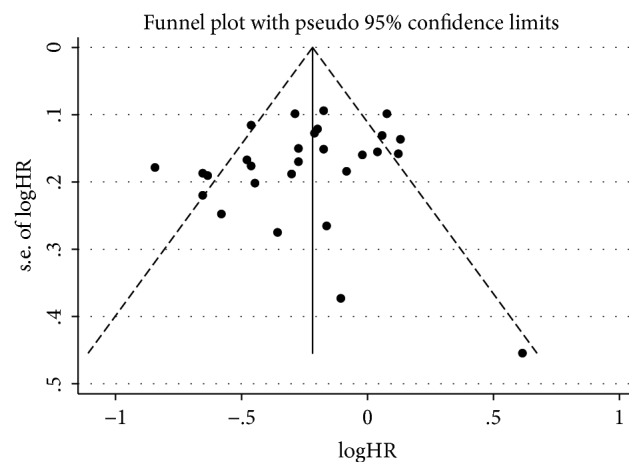
The publication bias analysis. Funnel plot of overall survival from both arms in included RCTs for the visual detection of systematic publication bias and small study effect. Each circle represents treatment effect expressed as the logarithm of the hazard ratio of overall survival in each trial plotted against standard error as a measure of study size. The diamond and the vertical line represent the pooled estimate from the meta-analysis.

A funnel plot of the primary outcome from younger arm, older arm, or their combination all showed slight asymmetry, which may indicate a possibility of publication bias across studies due to the limited number of included articles (Figure 3; Supplementary Figure 12A and 12B).

Similarly, just alike the older arm, younger arm revealed no obvious statistically significance in the Egger's test (*P=0.080*; Supplementary Figure 13A), and Begg's test (*P=0.193*; Supplementary Figure 12B), which means that there also exists none of the influence from publication bias. Interestingly, both the Egger's test (*P =0.761*; Supplementary Figure 13B) and the Begg's test (*P = 0.444*; Supplementary Figure 12A) showed that there was nothing to do with publication bias in older arm. What is more, neither the Egger's test (*P =0.245*; Supplementary Figure 13C) nor the Begg's test (*P = 0.186*; Supplementary Figure 12C) showed a statistically significance association between the study effects and the study size when both arms are taken into consideration together.

### 3.8. Sensitivity Analysis

To estimate the influence of single study on overall results of meta-analysis, we conducted sensitivity analysis as presented (Supplementary Figure 15A, 15B and 15C); the analysis showed that the pooled results were not significantly changed after deleting each trial, which confirmed the rationality and reliability of our meta-analysis.

## 4. Discussion

Based on the previous research, we confirmed that age factor takes effect in the immune system and interplay with ICIs. Hence, we conducted a systematic review and meta-analysis of advanced or metastatic lung cancer immunological studies to compare the clinical efficacy of ICIs between younger and older patients.

As far as we know, this is the first systematic review and meta-analysis to assess the efficacy of ICIs in patients with advanced or metastatic lung cancer due to patient's age. In a previous meta-analysis accomplished by Tomohiro and colleagues [37], they included only nine RCTs covering several cancer types and did not show a specific interaction between the ICIs and patient's age with advanced or metastatic lung cancer, because the analysis only included two out of nine trials for advanced or metastatic lung cancer so that it was not sufficient enough to do a subgroup analysis in terms of advanced or metastatic lung cancer. However, unlike the former research, our systematic review and meta-analysis, which included 12 RCTs consisting of 8176 participants, showed that ICIs, with or without other immunological or platinum-based chemotherapy drugs, can improve OS for patients in both age arms with advanced or metastatic lung cancer. Moreover, patients younger than 65 yr provided a comparable benefit from those ICIs versus control treatments than do the patients older than 65 yr. Based on existing knowledge in this area, hyperprogressive diseases have been reported to be associated with age older than 65 yr [38], which mainly occurs in a fraction of patients developing accelerated disease progression under ICIs and always leads to a dramatically reduction in OS [39]. Simultaneously, the accelerative progressive observed in cancer patients is evidently on account of tumor cell genetic alterations and oncogenic signaling activation, which could trigger inflammation, angiogenesis, or metabolism modification, and ultimately led to immune resistance or escape [40].

There was moderate to high heterogeneity across all included trials. The heterogeneity mainly resulted from the pathogenic type, cancer histotype, line of treatment, blind method and type of ICIs given in the intervention group. Therefore, we also did subgroup analysis, sensitivity analysis, and meta-regression analysis to figure out the possible source of heterogeneity. Firstly, our results demonstrated an increased efficacy of ICIs in younger patients versus older patients in NSCLC, but still without reliable outcomes in SCLC, for which ICIs plus platinum-based chemotherapy seems not to be clinical effective in younger arm on account of the specific pathogenic type. On the other hand, only about 15% to 20% of the advanced or metastatic lung cancer cases belong to SCLC, whereas NSCLC cancer approximately accounts for 80% to 85%. Hence, the research on SCLC is often restricted by the amount of cases and lack of stabilization, so the obtained results are correspondingly lack of conviction. Secondly, to lucubrate the benefit with regard to the class of ICIs administrated, we applied twelve out of thirteen RCTs done only in anti-PD-1/PD-L1 or anti-CTLA-4 inhibitors for a subgroup analysis. The subgroup analysis compared ten trials done in anti-PD-1/PD-L1 inhibitors with two trials done in anti-CTLA-4 inhibitors, and only the former showed a significant improvement in OS. It revealed that PD-1/PD-L1 is a correlated target point in both age arms. However, the pooled outcome in CTLA-4 inhibitor has no statistical significant difference, which is likely to be connected with the vastly different size observed between patients with anti-PD-1/PD-L1 and anti-CTLA-4 inhibitors. Thirdly, in accordance with the setting line of immunotherapy, first-line or subsequent-line, subgroup analyses were performed to reveal the sources of heterogeneity and their specific influences in older patients, but not in younger patients. For older patients, subgroup analysis confirmed that ICIs applied in subsequent-line settings could increase the OS with mild heterogeneity. And it is difficult to explain the heterogeneity in younger arms, whether in first-line or subsequent-line. Therefore, there is insufficient evidence that could prove that the line of treatment plays an important role in age-related immunotherapy. Moreover, the negative results may be on account of the imbalanced enrollment in first-line and subsequent-line patients. Fourthly, although subgroup analysis failed to reveal that there exists statically obvious difference from masking method both in younger and older arm, meta-regression analysis was conducted to exploit links between heterogeneous data from sources in two arms. It suggested that our results regarding OS should be interpreted with very caution in view of masking method. What is more, after taking histotype subgroup into consideration in older arm, it has been observed that patients with squamous-cell cancer did not significantly alter survival benefit of ICIs, which was opposite to the result in the non-squamous subset. Additionally, our results were strengthened by the meta-regression analysis that there existed a potential heterogeneity for OS, so it might have a certain influence on the reliability of histotype analysis. But pooled data were insufficient to draw definite conclusions, because only three in non-squamous and two in squamous studies performed the OS analysis in terms of histotype. Last but not least, after subdividing the older arm with 75 yr as a further cut-off age, surprisingly, we were incapable of observing the same conclusions whether in ≥65 and <75 yr or ≥75 yr subgroup, which is completely opposed to the pooled data in older arm. We thought that this opposite result was on account of the highest mortality rate due to decreased physiologic reserve and increased risks of iatrogenic toxicities or death. Beyond that, in view of immune system, it might be mainly because of immunosenescence [41] including thymic atrophy [42], decreases in native T cells, and increases in memory T cells with residual functionality [43], as well as decreases in antigen recognition [44]. However, interesting data from Curtis and colleagues suggest that patients over the age of 60 responded more efficiently to anti-PD-1 inhibitor, and likelihood of response to anti-PD-1 increased with age. This research came to conclude the diametrically opposition to ours; that might be because of several reasons as follows. On one hand, they generate and test the opposed conclusion in mice, instead of conducting clinical trials with the same parameters from which to draw relative comparisons. On the other hand, the type of cancer is different from our study population, and their observation that Treg numbers in aged spleens are much higher than those of young, whereas intratumoral Tregs numbers are lower, could be attributed to differences in Tregs populations, or their different ability to attack the tumor. Thus, distinguishing between the several different types of Tregs, and their distribution across a spectrum of aging and cancer, is badly in need.

Our study has several potential limitations as follows. Firstly, our meta-analysis is based on published clinical trials rather than on individual patients' data, which could provide more accurate age-related outcomes on the efficacy of ICIs. Secondly, the impact of patients' age should be taken into consideration in the assessment of both side effects and clinical benefit. Thirdly, our pooled results have shown that there is quite substantial heterogeneity among the included studies, and it is possible to have a close connection with the different types of ICIs, cancer histotype, pathogenic type, and other relevant factors included in our study. Therefore, we had minimized its influence as much as possible by using random-effect model, as well as conducting prespecified subgroup and sensitivity analysis. For another, the pooled population was approximately several times the size for NSCLC patients than for SCLC patients and PD-1 inhibitor than for CTLA-4 inhibitor so that uncertain outcomes were generated by the size of the difference, which made it impossible to obtain specific conclusions. Hence, researches who are conducting immune-related studies should guarantee more clinical trials for SLCL, so that they avoid mistakenly drawing a general conclusion mainly from patients with NSCLC. Next, we observed different results between older arm itself and its subsets; hence we should not ignore the further cut-off in older arm, which could have a reversed affect in the immune response to ICIs. Furthermore, we performed the data analysis by using HR and 95% CI in combination from younger and older arm, respectively, rather than the figure for a direct and quantitative comparison between two arms. Finally, the patients in this study were selective with good performance status who were taken into the clinical trials at different academic centers. Because of this, it is possible for the observed results that may not be entirely objective to patients rolled in clinical trials.

## 5. Conclusion

Broadly speaking, this systematic review and meta-analysis came to the main conclusion being summarized as follows: ICIs have the ability to significantly prolong OS whether in younger or older arm, but the magnitude of this clinical benefit is age-dependent to a certain extent. Generally speaking, our analysis revealed that older patients received almost similar benefits with younger patients when treated with ICIs, but there is still a comparable OS benefit for ICIs in younger arm (<65 yr) than older arm (≥65 yr). Conversely, we also drew a statically significant conclusion that older patients failed to acquire benefit from ICIs when subdivided with a further cut-off of 75 yr. Specifically speaking, younger patients received a better OS benefit in advanced or metastatic lung cancer patients with anti-PD-1/PD-L1 inhibitors, Pembrolizumab or Atezolizuma used, NSCLC, squamous and subsequent-line setting, while older patients had an advantage over other conventional therapies in patients with advanced or metastatic lung cancer, especially for those patients with anti-PD-1/PD-L1 inhibitors, NSCLC, non-squamous, Pembrolizumab, Atezolizumab or Ipilimumab used and subsequent-line setting. Therefore, more future researches are supposed to focus on improving the efficacy of ICIs in older patients with further cut-off in terms of different physiological age. In addition to this, older patients should be positively encouraged to take part in more clinical trials of these immunological agents in the future. Moreover, the role of different immunotherapeutic target, histotype or pathology of lung cancer, treatment line in younger and older patients with advanced or metastatic lung cancer treated with ICIs is still unclear and also warrants further exploration.

## Figures and Tables

**Table 1:** Characteristics of studies included in this meta-analysis.

Author, year	Phase	Pathology	Line	Treatment groups	No. of Patients	Median age, years	No. of younger (%)	No. of older (%)	Follow-up (months)	Overall survival		
										Overall HR (95% CI)	HR (95% CI) for younger	HR (95% CI) for older
Brahmer (2015)	3	NSCLC	>1	Nivolumab vs Docetaxel	272	63.0 (39-85)	152 (56%)	120 (44%)	11.0 months†	0.63 (0.48-0.82)	<65 yr: 0.62 (0.44-0.89)	A (≥65 and <75 yr): 0.51 (0.32-0.82) B (≥75 yr): 1.76 (0.77-4.05)

Borghaei (2015)	3	NSCLC	>1	Nivolumab vs Docetaxel	582	62.0 (21-85)	339 (58%)	243 (42%)	17.2 months	0.75 (0.62-0.91)	<65 yr: 0.81 (0.62-1.04)	A (≥65 and <75 yr): 0.63 (0.45-0.89) B (≥75 yr): 0.90 (0.43-1.87)

Herbst (2015)	2/3	NSCLC	>1	Pembrolizumab (2 mg/kg) vs Pembrolizumab (10 mg/kg) vs Docetaxel	1033	62.5 (54-70)	604 (58%)	429 (42%)	13.1 months	0.67 (0.56–0.80)	<65 yr: 0.63 (0.50-0.79)	≥65 yr: 0.76 (0.57–1.02)

Reck (2016)	3	SCLC	1	Ipilimumab + EP vs Placebo + EP	954	62.5 (36-85)	576 (60%)	378 (40%)	10.5 vs10.2 months	0.94 (0.81-1.09)	<65 yr: 1.08 (0.90-1.31)	A (≥65 and <75 yr): 1.14 (0.87 to 1.49) B (≥75 yr): 0.70 (0.40 to 1.20)

Carbone (2017)	3	NSCLC	1	Nivolumab vs Investigator's choice of platinum doublet chemotherapy	541	64.0 (29-89)	281 (52%)	260 (48%)	18.0 months	1.08 (0.87–1.34)	<65 yr: 1.13 (0.83-1.54)	≥65 yr: 1.04 (0.77–1.41)

Govindan (2017)	3	NSCLC	1	Ipilimumab + PC vs Placebo +PC	749	64.0 (28-85)	380 (51%)	369 (49%)	12.5 vs 11.8 months	0.91 (0.77–1.07)	<65 yr: 0.82 (0.64-1.04)	A (65 and ≤ 74 yr): 1.06 (0.81-1.37) B (≥75 yr): 0.85 (0.51-1.43)

Gandhi (2018)	3	NSCLC	>1	Pembrolizumab + Pemetrexed + PBC vs Placebo + Pemetrexed + PBC	616	64.0(34-84)	312 (51%)	304 (49%)	10.5 months	0.49 (0.38–0.64)	<65 yr: 0.43 (0.31-0.61)	≥65 yr: 0.64 (0.43–0.95)

Paz-Ares (2018)	3	NSCLC	1	Pembrolizumab + ICC vs Placebo + ICC	559	65.0 (29-88)	254 (45%)	305 (55%)	7.8 months	0.64 (0.49–0.85)	<65 yr: 0.52 (0.34-0.80)	≥65 yr: 0.74 (0.51–1.07)

Horn (2018)	3	SCLC	1	Atezolizumab + CE vs Placebo + CE	403	64.0 (26–90)	217 (54%)	186 (46%)	13.9 months	0.70 (0.54–0.91)	<65 yr: 0.92 (0.64-1.32)	≥65 yr: 0.53 (0.36–0.77)

Antonia (2018)	3	NSCLC	>1	Durvalumab vs Placebo	713	64.0 (23-90)	391 (55%)	322 (45%)	25.2 months	0.68 (0.54-0.86)	<65 yr: 0.62 (0.44-0.86)	≥65 yr: 0.76 (0.55–1.06)

Barlesi (2018)	3	NSCLC	>1	Avelumab vs Docetaxel	529	63.5 (56-70)	279 (53%)	250 (47%)	18.9 months	0.90 (0.73–1.12)	<65 yr: 0.84 (0.63-1.13)	≥65 yr: 0.98 (0.71–1.34)

Fehrenbacher (2018)	3	NSCLC	>1	Atezolizumab vs Docetaxel	1225	63.5 (25-85)	661 (54%)	564 (46%)	26.0 months	0.80 (0.70-0.92)	<65 yr: 0.84 (0.79-1.01)	≥65 yr: 0.75 (0.61-0.91)

*Abbreviations*. NSCLC, non-small cell lung cancer; SCLC, small cell lung cancer. †Represents minimum follow-up time between the start of the study (the first visit of the first enrolled patient) and the end of the study (the last visit of the last enrolled patient). EP=Etoposide plus Platinum. PC=Paclitaxel plus Carboplatin. PDC=Platinum doublet chemotherapy. PBC=Platinum-based chemotherapy. ICC= investigator's choice of chemotherapy. CE= Carboplatin plus Etoposide.

**Table:** (a) Analysis of age-specific pooled hazard ratios and 95%CI of overall survival for younger patients assigned to intervention treatment, compared with those assigned to control treatment, by subgroup

Analysis	N	Random-effects model		Fixed-effects model		Heterogeneity	
		HR (95% CI)	*P*	HR (95% CI)	*P*	I^2^	*P*
*Class of immune checkpoint inhibitor*	12	0.75 (0.65, 0.87)	≤0.001	0.80 (0.75, 0.86)	≤0.001	75.1%	≤0.001
PD-1/PD-L1	10	0.71 (0.60, 0.84)	≤0.001	0.76 (0.70, 0.82)	≤0.001	72.2%	≤0.001
CTLA-4	2	0.95 (0.73, 1.25)	0.718	0.97 (0.84, 1.13)	0.731	67.7%	0.079

*Type of immune checkpoint inhibitor*	10	0.75 (0.63, 0.90)	0.001	0.81 (0.75, 0.87)	≤0.001	78.4%	≤0.001
Nivolumab	3	0.79 (0.53, 1.18)	0.249	0.82 (0.69, 0.98)	0.028	79.2%	0.008
Pembrolizumab	3	0.54 (0.42, 0.68)	≤0.001	0.55 (0.46, 0.66)	≤0.001	42.0%	0.178
Atezolizumab	2	0.85 (0.75, 0.95)	0.005	0.85 (0.75, 0.95)	0.005	0.0%	0.641
Ipilimumab	2	0.95 (0.73, 1.25)	0.718	0.97 (0.84, 1.13)	0.731	67.7%	0.079

*Pathology*	12	0.75 (0.65, 0.87)	≤0.001	0.80 (0.75, 0.86)	≤0.001	75.1%	≤0.001
Small cell lung cancer	2	1.04 (0.88, 1.23)	0.613	1.04 (0.88, 1.23)	0.613	0.0%	0.441
Non-small cell lung cancer	10	0.71 (0.60, 0.83)	≤0.001	0.76 (0.70, 0.82)	≤0.001	71.6%	≤0.001

*Histotype*	5	0.62 (0.47, 0.81)	≤0.001	0.66 (0.58, 0.76)	≤0.001	72.1%	0.006
Squamous	3	0.63 (0.45, 0.88)	0.006	0.68 (0.56, 0.81)	≤0.001	64.9%	0.058
Non-squamous	2	0.60 (0.32, 1.11)	0.102	0.64 (0.52, 0.79)	≤0.001	88.2%	0.004

*Line*	12	0.75 (0.65, 0.87)	≤0.001	0.80 (0.75, 0.86)	≤0.001	75.1%	≤0.001
First-line	5	0.89 (0.71, 1.12)	0.329	0.94 (0.84, 1.06)	0.332	66.8%	0.017
Subsequent line	7	0.67 (0.56, 0.81)	≤0.001	0.74 (0.68, 0.80)	≤0.001	72.0%	0.002

*Masking*	12	0.75 (0.65, 0.87)	≤0.001	0.80 (0.75, 0.86)	≤0.001	75.1%	≤0.001
Double-blind	6	0.71 (0.53, 0.95)	0.022	0.80 (0.71, 0.90)	≤0.001	83.0%	≤0.001
Open-label	6	0.78 (0.66, 0.93)	0.005	0.80 (0.73, 0.87)	≤0.001	65.8%	0.012

**Table:** (b) Analysis of age-specific pooled hazard ratios and 95%CI of overall survival for older patients assigned to intervention treatment, compared with those assigned to control treatment, by subgroup

Analysis	N	Random-effects model		Fixed-effects model		Heterogeneity	
		HR (95% CI)	*P*	HR (95% CI)	*P*	I^2^	*P*
*Class of immune checkpoint inhibitor*	12	0.81 (0.72, 0.92)	0.001	0.82 (0.76, 0.90)	≤0.001	48.8%	0.015
PD-1/PD-L1	10	0.76 (0.67, 0.87)	≤0.001	0.76 (0.69, 0.84)	≤0.001	37.1%	0.094
CTLA-4	2	1.02 (0.86, 1.21)	0.783	1.02 (0.87, 1.21)	0.777	0.7%	0.388

*Type of immune checkpoint inhibitor*	10	0.80 (0.69, 0.93)	0.003	0.82 (0.74, 0.89)	≤0.001	53.4%	0.009
Nivolumab	3	0.83 (0.59, 1.18)	0.304	0.82 (0.67, 0.99)	0.042	60.9%	0.037
Pembrolizumab	3	0.72 (0.59, 0.88)	0.001	0.72 (0.59, 0.88)	0.001	0.0%	0.782
Atezolizumab	2	0.66 (0.47, 0.91)	0.013	0.70 (0.58, 0.83)	≤0.001	60.2%	0.113
Ipilimumab	2	1.02 (0.86, 1.21)	0.783	1.02 (0.87, 1.21)	0.777	0.7%	0.388

*Pathology*	12	0.81 (0.72, 0.92)	0.001	0.82 (0.76, 0.90)	≤0.001	58.8%	0.015
Small cell lung cancer	2	0.76 (0.45, 1.29)	0.313	0.86 (0.70, 1.05)	0.132	81.8%	0.004
Non-small cell lung cancer	10	0.82 (0.72, 0.92)	0.001	0.82 (0.74, 0.90)	≤0.001	33.9%	0.111

*Histotype*	5	0.79 (0.64, 0.98)	0.030	0.80 (0.69, 0.93)	0.003	47.2%	0.066
Squamous	3	0.87 (0.65, 1.17)	0.347	0.89 (0.74, 1.07)	0.207	53.9%	0.070
Non-squamous	2	0.66 (0.52, 0.84)	0.001	0.66 (0.52, 0.84)	0.001	0.0%	0.678

*Line*	12	0.81 (0.72, 0.92)	0.001	0.82 (0.76, 0.90)	≤0.001	48.8%	0.015
First-line	5	0.87 (0.71, 1.07)	0.193	0.92 (0.81, 1.04)	0.182	59.0%	0.023
Subsequent line	7	0.76 (0.66, 0.86)	≤0.001	0.76 (0.68, 0.85)	≤0.001	19.0%	0.274

*Masking*	12	0.81 (0.72, 0.92)	0.001	0.82 (0.76, 0.90)	≤0.001	48.8%	0.015
Double-blind	6	0.80 (0.66, 0.98)	0.029	0.84 (0.75, 0.95)	0.007	57.9%	0.020
Open-label	6	0.81 (0.69, 0.97)	0.018	0.80 (0.72, 0.90)	≤0.001	43.4%	0.089

*NoteFoot*. The p value for heterogeneity is from the I^2^ test comparing the interaction HRs across subgroups including class of ICI, type of ICI, pathology, histotype, line of treatment, masking method, and represents heterogeneity within each subgroup. PD-1/PD-L1= programmed cell death-1/ programmed cell death-Ligand 1. CTLA4=cytotoxic T-lymphocyte protein 4.

## Supplementary Materials

Supplementary Materials Supplementary Tables Supplementary Table 1 -Further Characteristics of the included trial Supplementary Figures Supplementary Figure 1 – Risk of bias graph Supplementary Figure 2 – Risk of bias summary Supplementary Figure 3 – Survival According to a further cut-off Supplementary Figure 4 – Subgroup analysis of class of ICIs Supplementary Figure 5 – Subgroup analysis of histotype Supplementary Figure 6 – Subgroup analysis of line of treatment Supplementary Figure 7 – Subgroup analysis of masking method Supplementary Figure 8 – Subgroup analysis of type of ICIs Supplementary Figure 9 – Subgroup analysis of pathology Supplementary Figure 10 – Meta-Regression Analysis from younger patients Supplementary Figure 11 – Meta-Regression Analysis from older patients Supplementary Figure 12 – Funnel plot from younger and older arms Supplementary Figure 13 – Egger's test from younger, older and both arms Supplementary Figure 14 – Begg's test from younger, older and both arms Supplementary Figure 15 – Sensitivity analysis from younger, older and both arms.
