# Supplementary material for: Comparison of Immune Checkpoint Inhibitors between Older and Younger Patients with Advanced or Metastatic Lung Cancer: A Systematic Review and Meta-Analysis
Source: Biomed Res Int. 2019 May 13;2019:9853701. doi: 10.1155/2019/9853701 (PMC6535828; doi:10.1155/2019/9853701)

**SUPPLEMENTARY MATERIAL**

**Supplementary Tables**

**Supplementary Table 1** -Further Characteristics of the included trial

**Supplementary Figures**

**Supplementary Figure 1 –** Risk of bias graph

**Supplementary Figure 2 –** Risk of bias summary

**Supplementary Figure 3 –** Survival According to a further cut-off

**Supplementary Figure 4 –** Subgroup analysis of class of ICIs

**Supplementary Figure 5 –** Subgroup analysis of histotype

**Supplementary Figure 6 –** Subgroup analysis of line of treatment

**Supplementary Figure 7 –** Subgroup analysis of masking method

**Supplementary Figure 8 –** Subgroup analysis of type of ICIs

**Supplementary Figure 9 –** Subgroup analysis of pathology

**Supplementary Figure 10 –** Meta-Regression Analysis from younger patients

**Supplementary Figure 11 –** Meta-Regression Analysis from older patients

**Supplementary Figure 12 –** Funnel plot from younger and older arms

**Supplementary Figure 13 –** Egger’s test from younger, older and both arms

**Supplementary Figure 14 –** Begg’s test from younger, older and both arms

**Supplementary Figure 15 –** Sensitivity analysis from younger, older and both arms

| Author, year | Masking | Stage | Histology | Number of male (%) | Number of female (%) |
| --- | --- | --- | --- | --- | --- |
|  |  |  |  |  |  |
| Brahmer (2015) | Open-label | 3/4 | Squamous | 208 (76%) | 64 (24%) |
| Borghaei (2015) | Open-label | 3/4 | Non-squamous | 319 (55%) | 263 (45%) |
| Herbst  (2015) | Open-label | 4 | No Category | 634 (61%) | 399 (39%) |
| Reck  (2016) | Double-blind | extensive | — | 643 (67%) | 312 (33%) |
| Carbone (2017) | Open-label | 4 | No Category | 332 (61%) | 209 (39%) |
| Govindan（2017） | Double-blind | 4 | Squamous | 635 (85%) | 114 (15%) |
| Gandhi (2018) | Double-blind | 4 | Non-squamous | 363 (59%) | 253 (41%) |
| Paz-Ares (2018) | Double-blind | 4 | Squamous | 455 (81%) | 104 (19%) |
| Horn  (2018) | Double-blind | extensive | — | 261 (65%) | 142 (35%) |
| Antonia (2018) | Double-blind | 3 | No Category | 500 (70%) | 213 (30%) |
| Barlesi  (2018) | Open-label | 3/4 | No Category | 367 (69%) | 162 (31%) |
| Fehrenbacher (2018) | Open-label | 3 | No Category | 758 (62%) | 467 (38%) |

**Supplementary Table1**- Further Characteristics of the included trial

**Supplementary Figure 1** - Risk of bias graph: Review authors' judgments about each risk of bias item presented as percentages across all included studies.


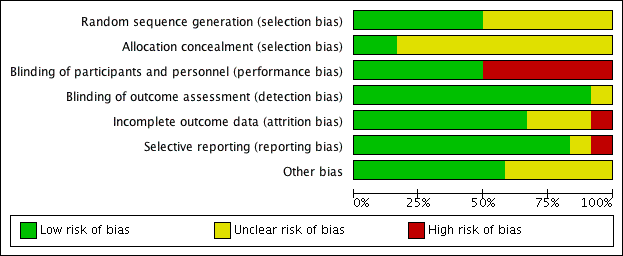


**Supplementary Figure 2** - Risk of bias summary: Review authors’ judgments about each risk of bias item for each included study.


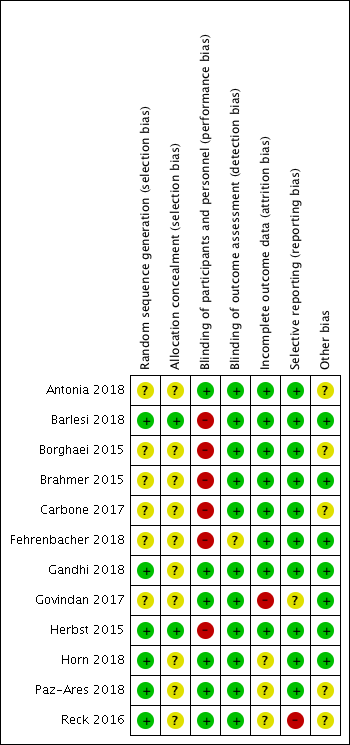


**Supplementary Figure 3** - Survival According to a further cut-off: subdivide the older arm with 75 yr as a further cut-off.

**
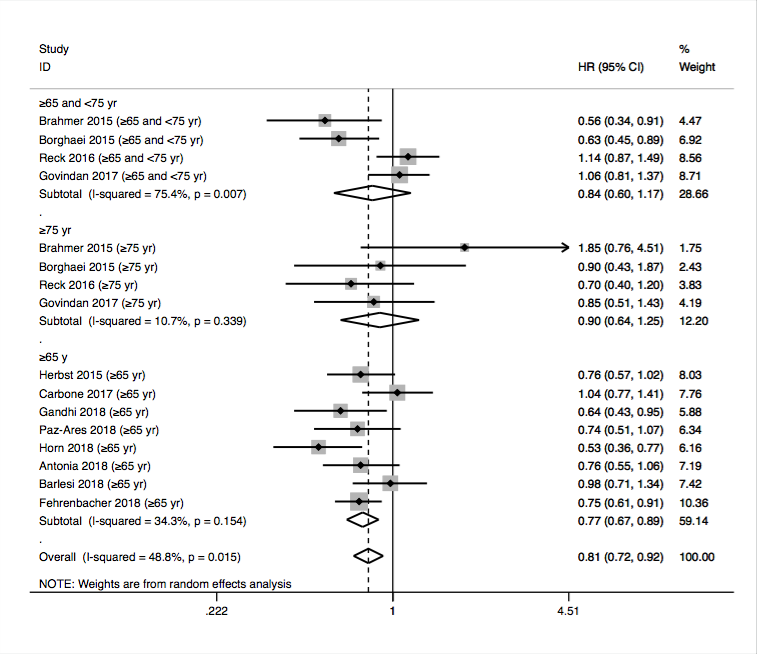
**

**Supplementary Figure 4** – Subgroup analysis of class of ICIs: Subgroup analysis of age-specific pooled hazard ratios and 95%CI of overall survival for younger (A) and older (B) patients assigned to intervention treatment, compared with those assigned to control treatment, by class of of ICIs.

**A**

**
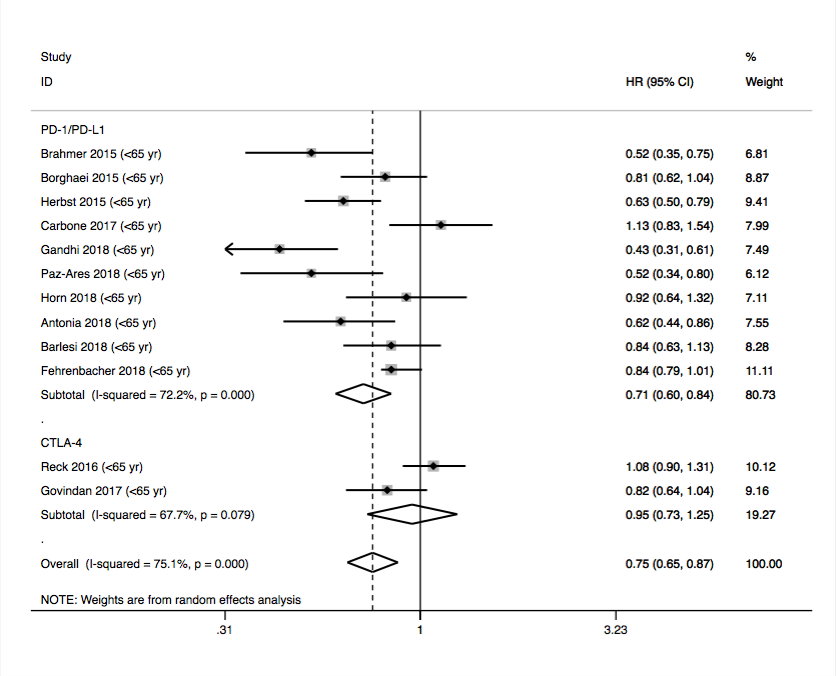
**

B


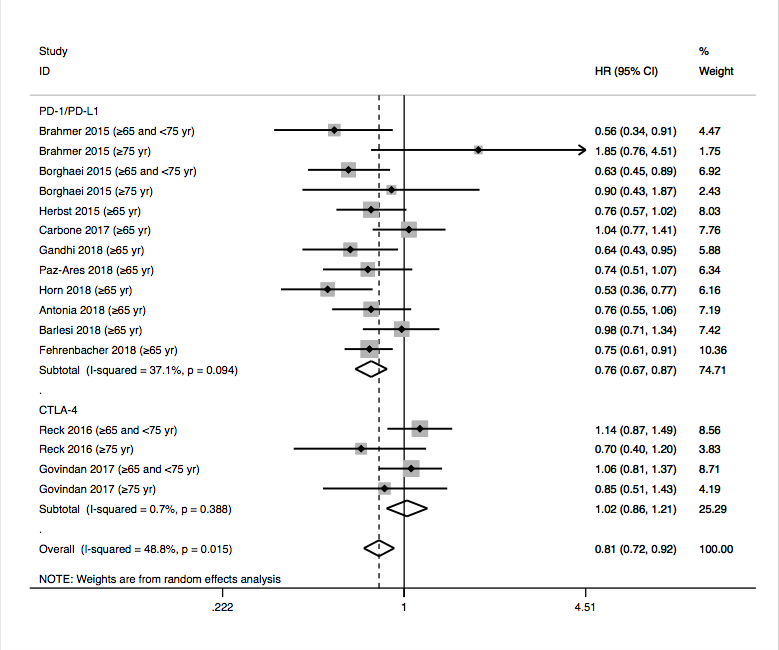


**Supplementary** **Figure 5** – Subgroup analysis of histotype: Subgroup analysis of age-specific pooled hazard ratios and 95%CI of overall survival for younger (A) and older (B) patients assigned to intervention treatment, compared with those assigned to control treatment, by histotype.

**A**

**
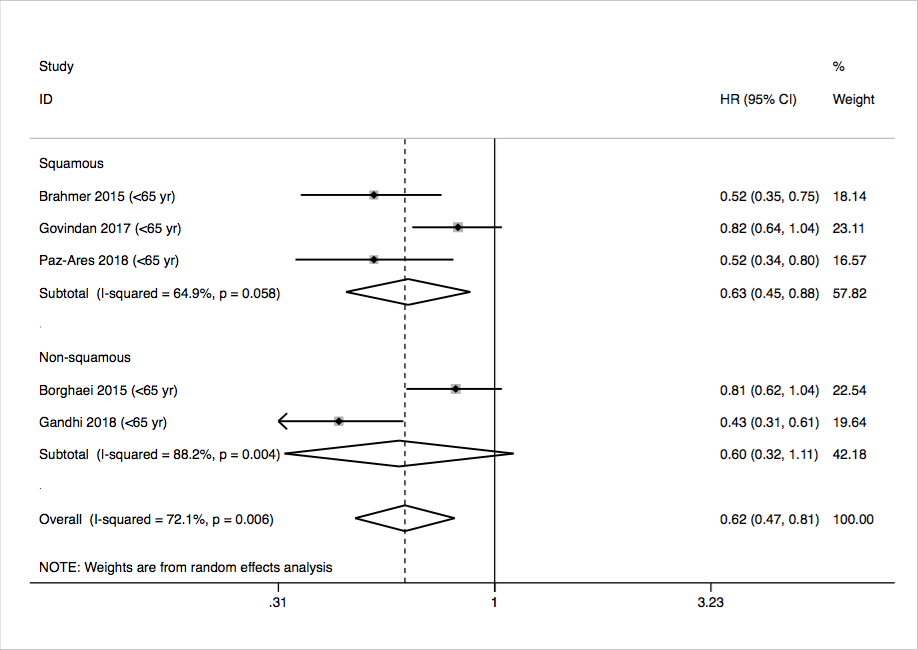
**

**B**

**
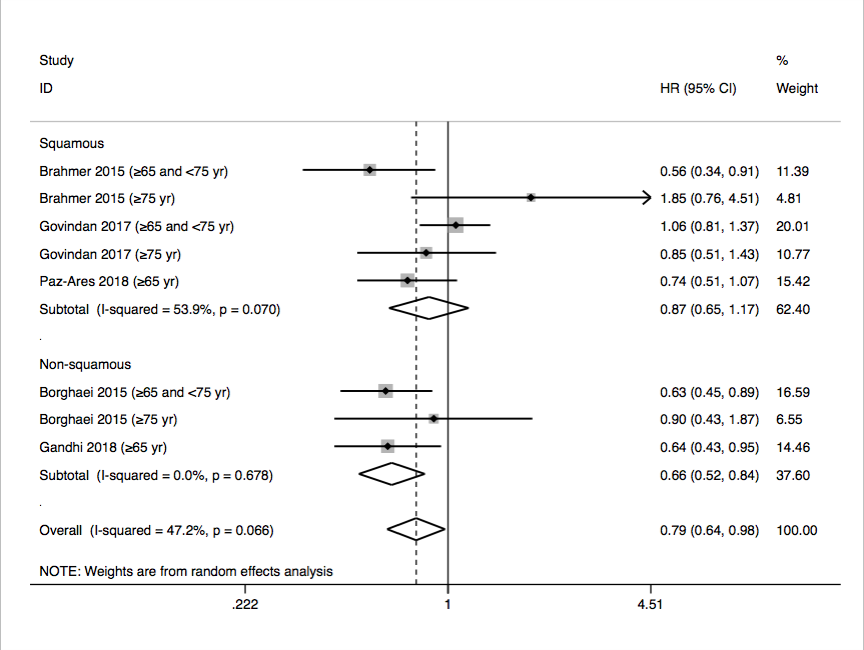
**

**Supplementary** **Figure 6** – Subgroup analysis of line of treatment: Subgroup analysis of age-specific pooled hazard ratios and 95%CI of overall survival for younger (A) and older (B) patients assigned to intervention treatment, compared with those assigned to control treatment, by line of treatment.

**A**

**
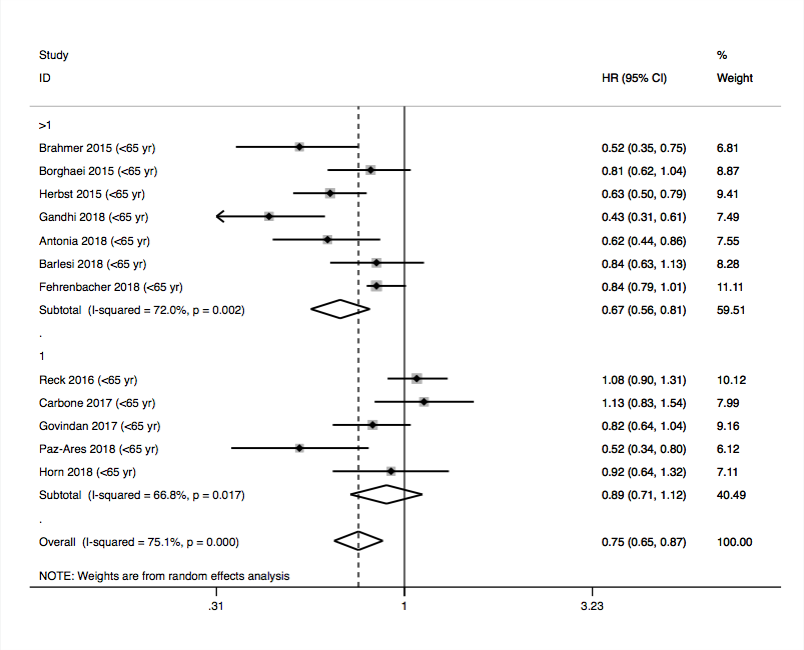
**

**B**

**
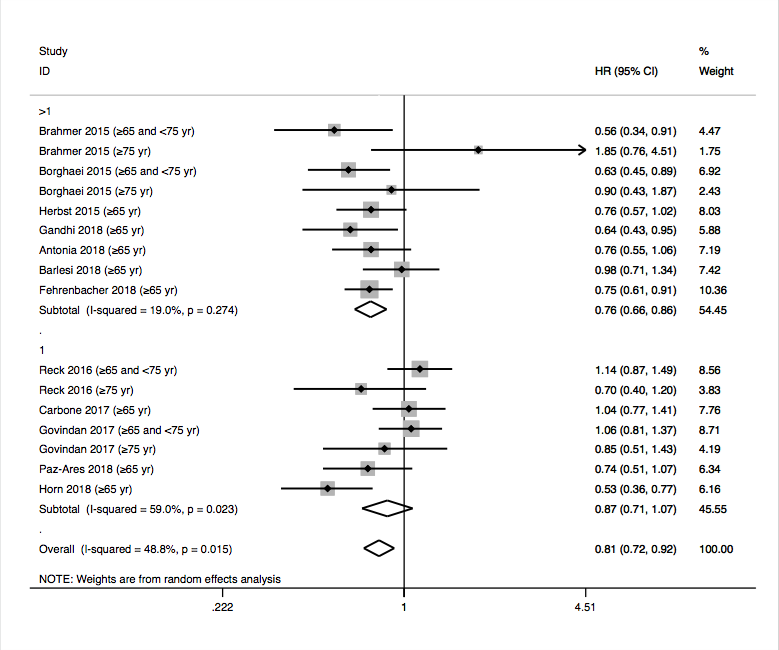
**

**Supplementary** **Figure 7** – Subgroup analysis of masking method: Subgroup analysis of age-specific pooled hazard ratios and 95%CI of overall survival for younger (A) and older (B) patients assigned to intervention treatment, compared with those assigned to control treatment, by masking method.

**A**

**
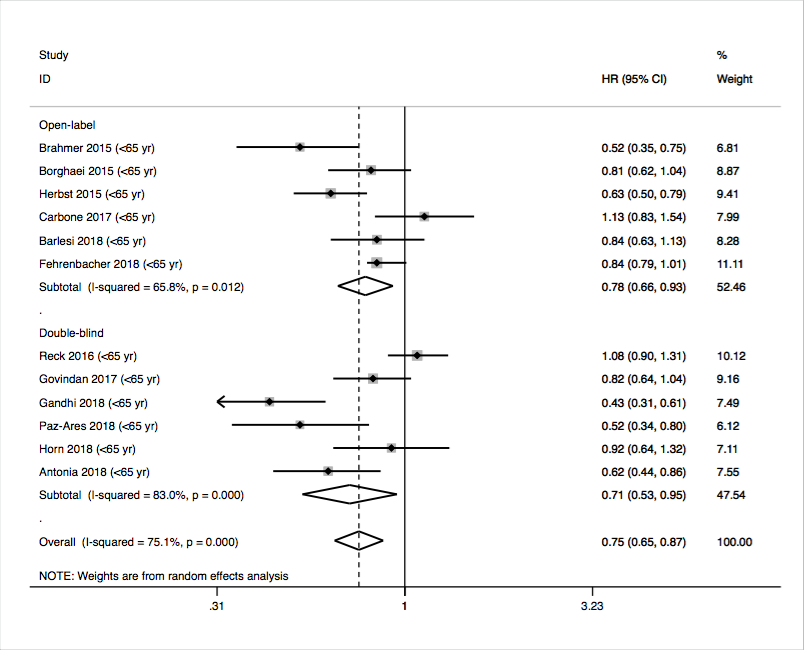
**

**B**

**
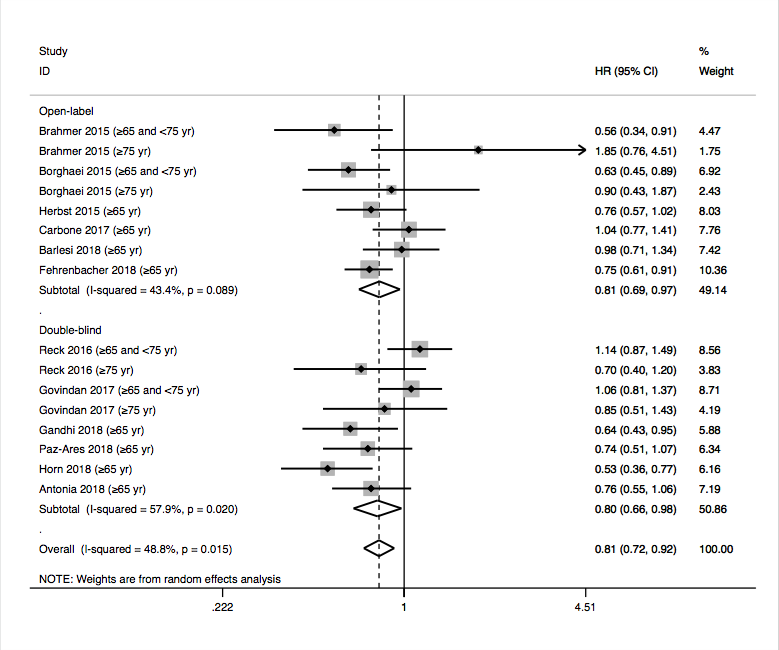
**

**Supplementary** **Figure 8** – Subgroup analysis of type of ICIs: Subgroup analysis of age-specific pooled hazard ratios and 95%CI of overall survival for younger (A) and older (B) patients assigned to intervention treatment, compared with those assigned to control treatment, by type of ICIs.

**A**

**
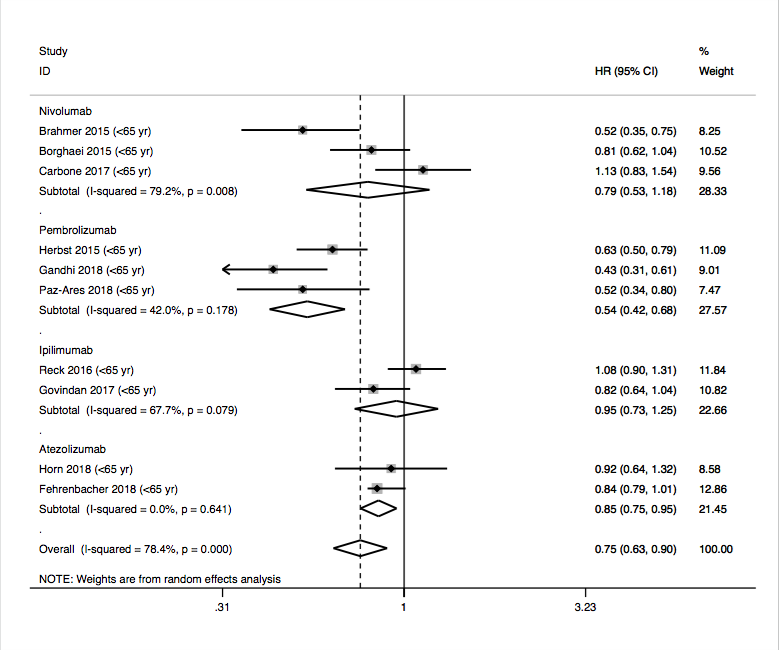
**

**B**

**
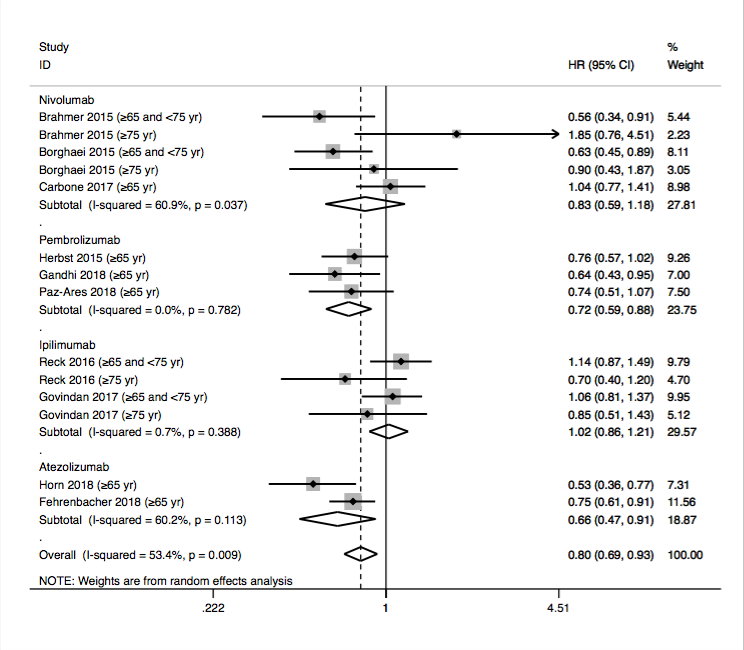
**

**Supplementary** **Figure 9** – Subgroup analysis of pathology: Subgroup analysis of age-specific pooled hazard ratios and 95%CI of overall survival for younger (A) and older (B) patients assigned to intervention treatment, compared with those assigned to control treatment, by pathology.

**A**

**
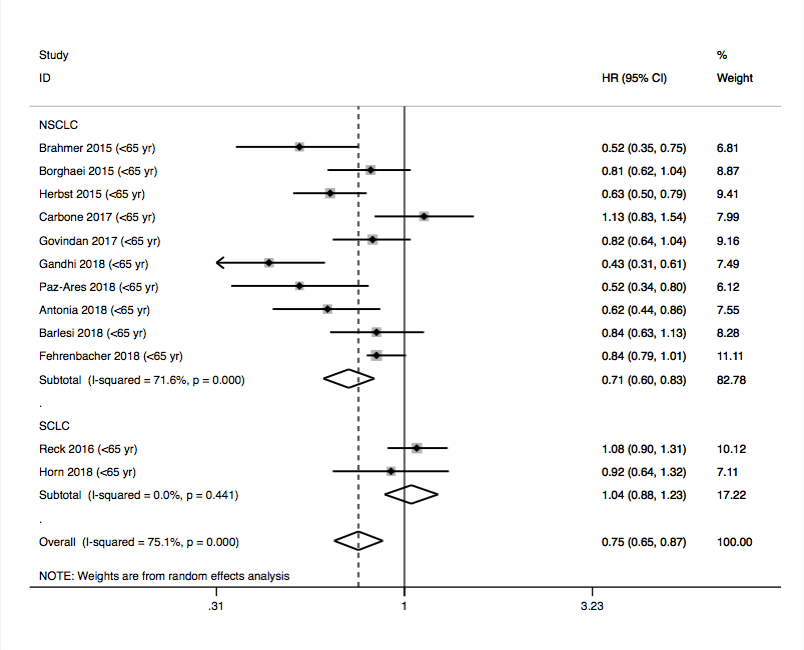
**

**B**

**
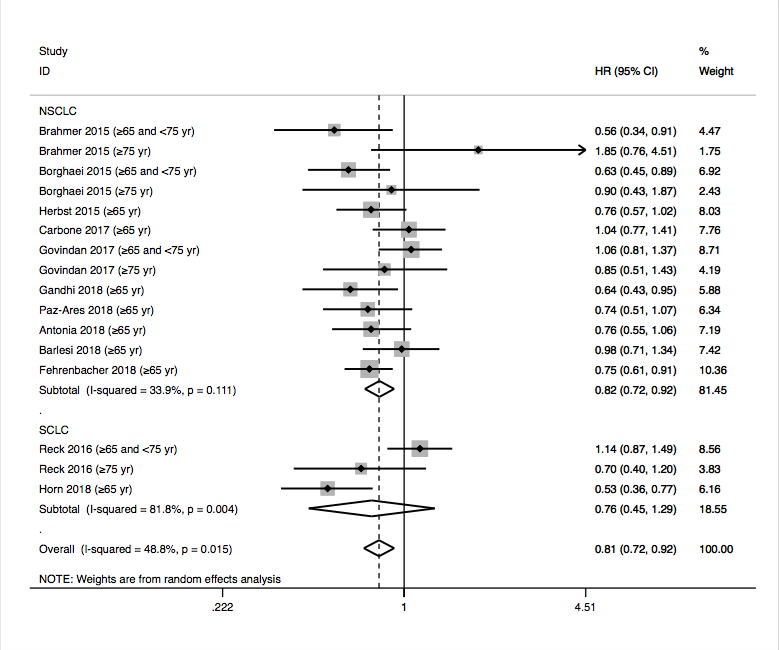
**

**Supplementary** **Figure 10** – Meta-Regression Analysis from younger patients: Meta-Regression Analysis of covariates as potential explanatory factors of heterogeneity from younger patients.

**
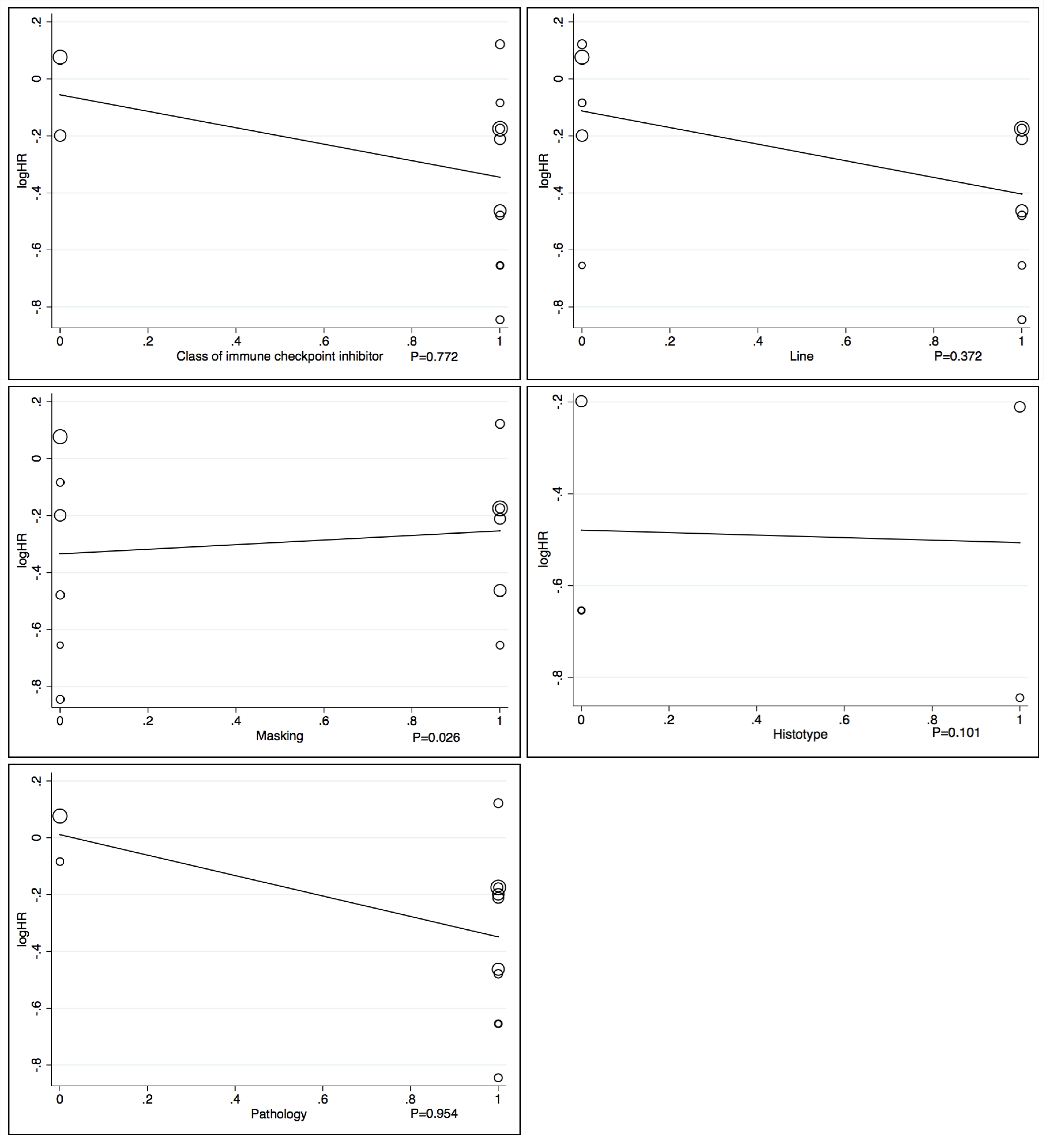
**

**Supplementary** **Figure 11** – Meta-Regression Analysis from older patients: Meta-Regression Analysis of covariates as potential explanatory factors of heterogeneity from older patients.

**
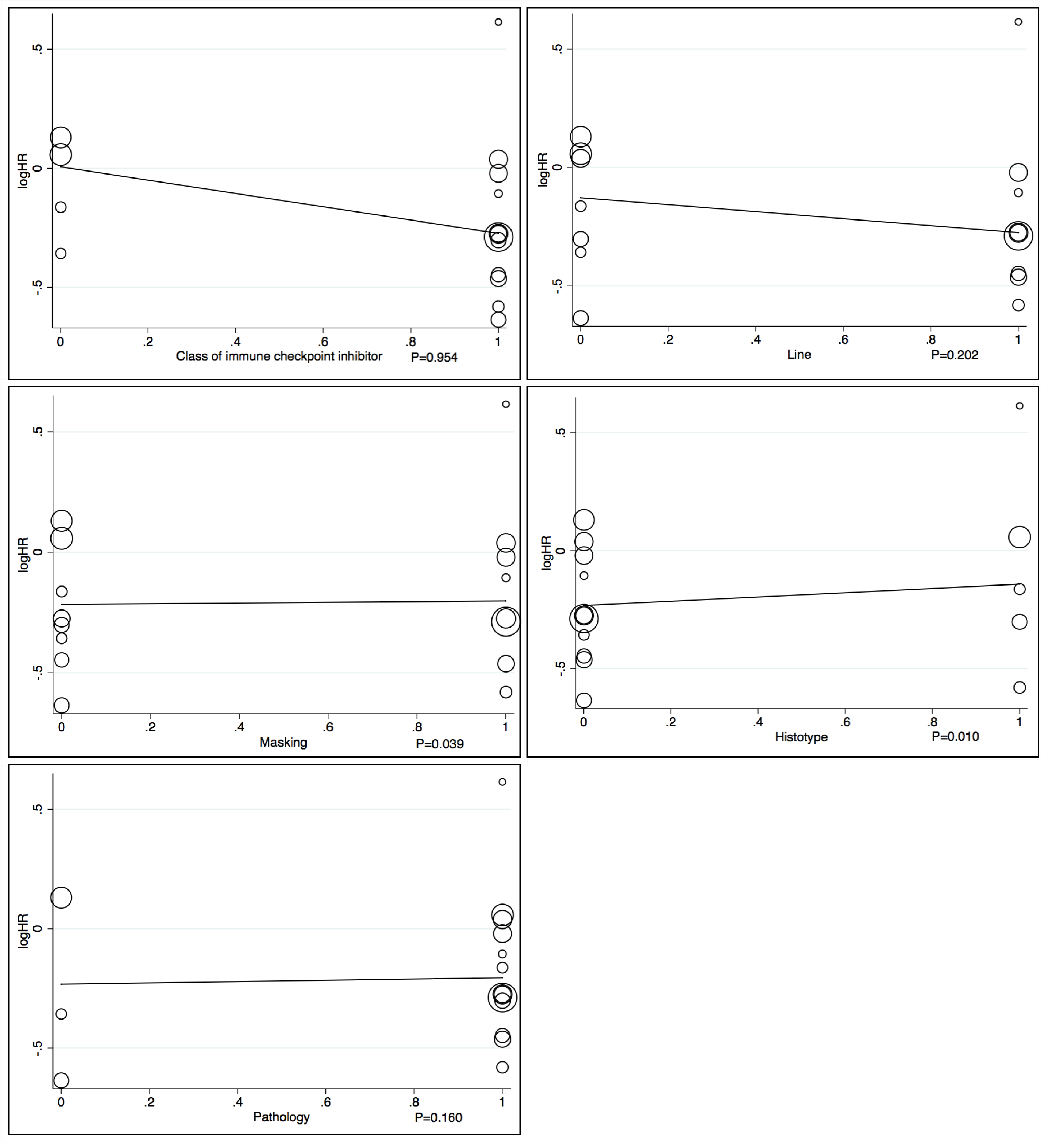
**

**Supplementary** **Figure 12** - Funnel plot from younger and older arms: Funnel plot of overall survival from younger (A) and older (B) arms in included RCTs for the visual detection of systematic publication bias and small study effect.

**A**


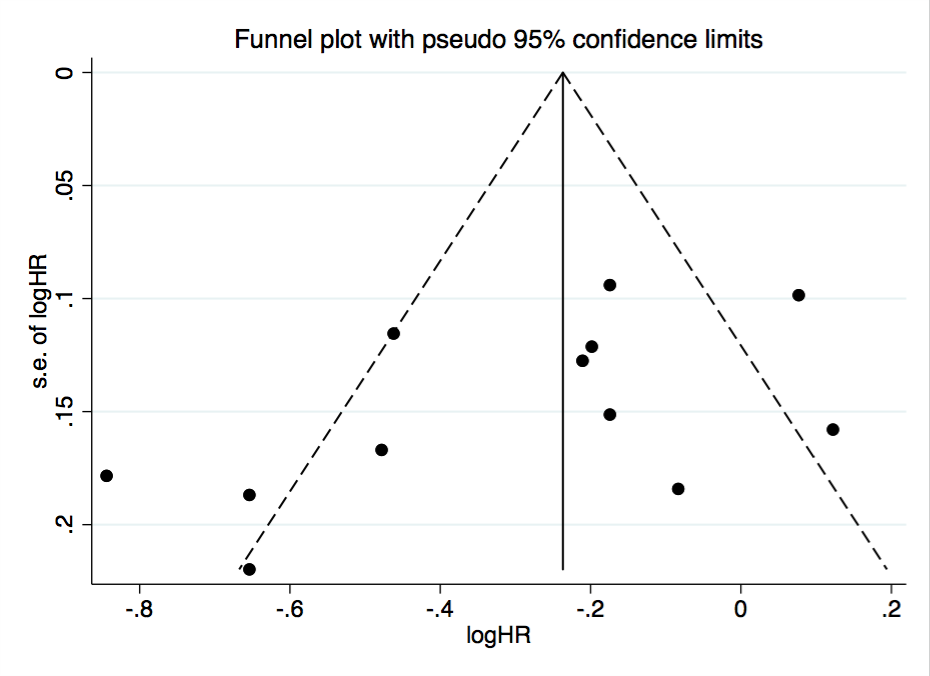


**B**

**
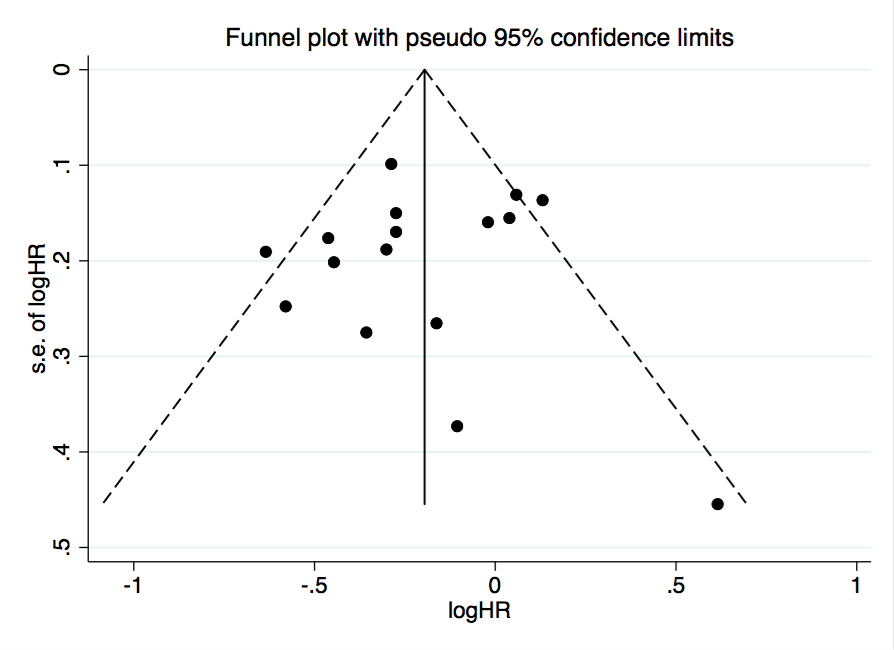
**

**Supplementary Figure 13** - Egger’s test from younger, older and both arms: Egger’s test of overall survival from younger (A), older (B) and both arms (C) in included RCTs for calculative detection of association between the study effects and the study size.

**A**


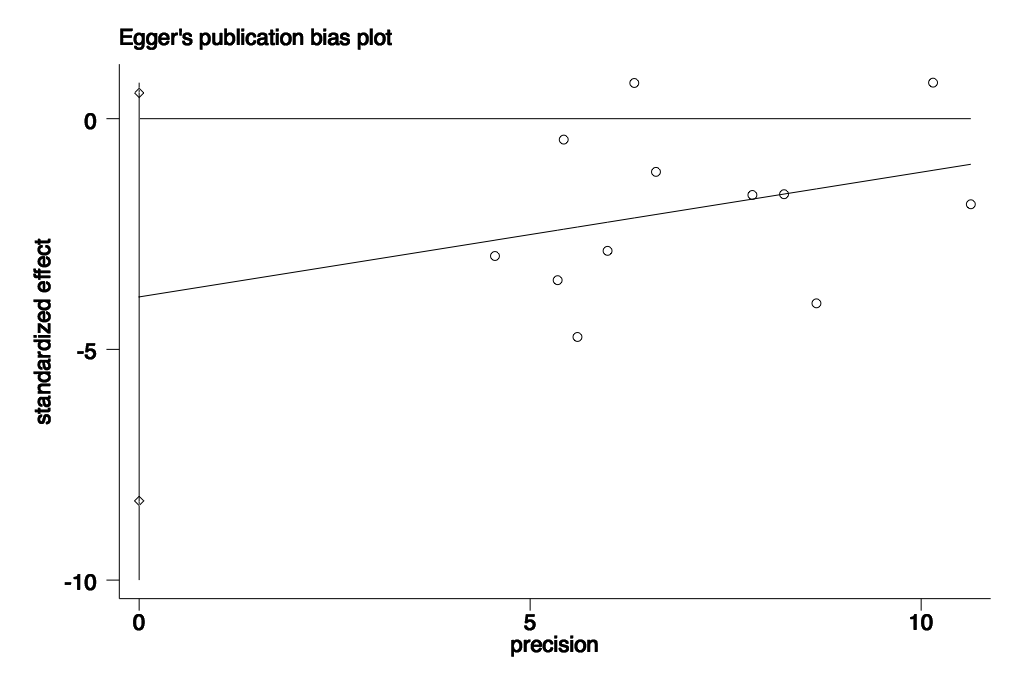


**B**


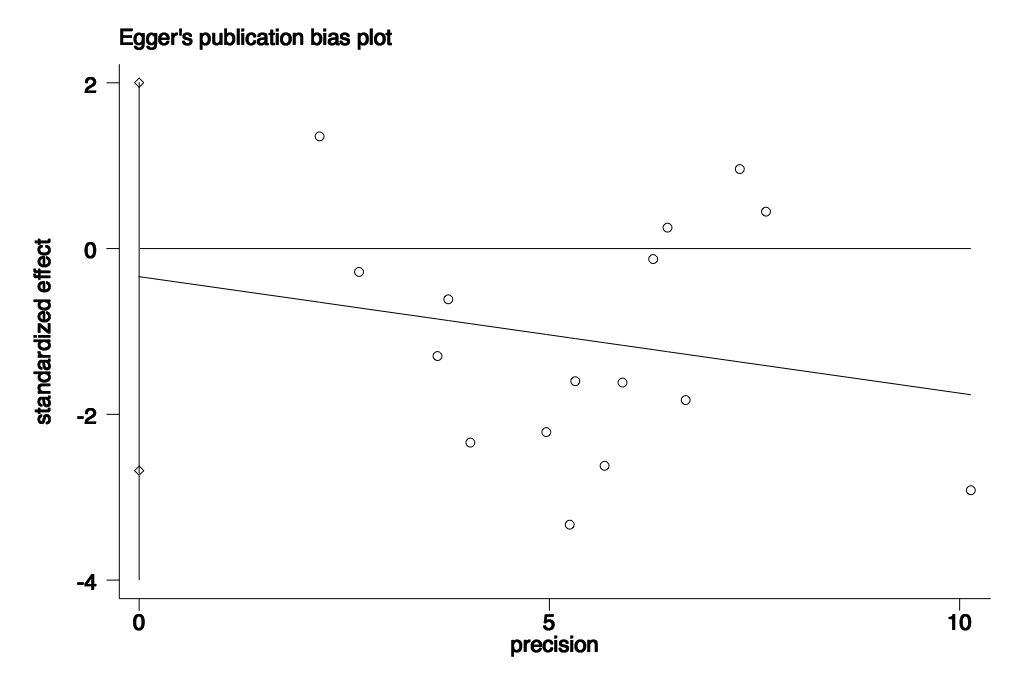


**C**

**
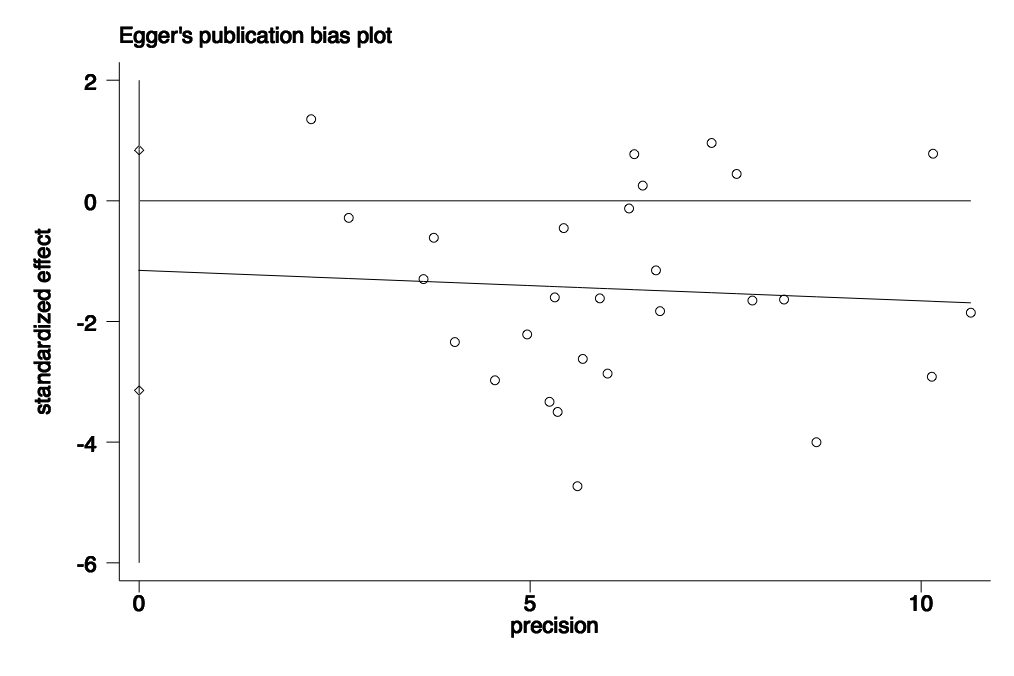
**

**Supplementary Figure 14** - Begg’s test from younger, older and both arms: Begg’s test of overall survival from younger (A), older (B) and both arms (C) in included RCTs for calculative detection of association between the study effects and the study size.

**A**

**
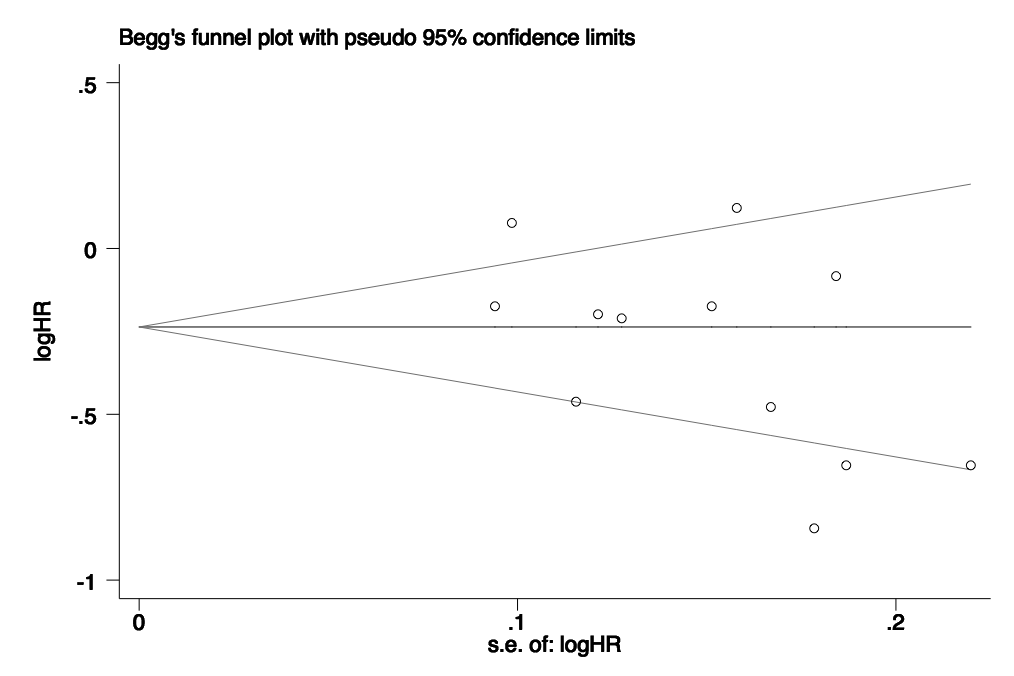
**

**B**


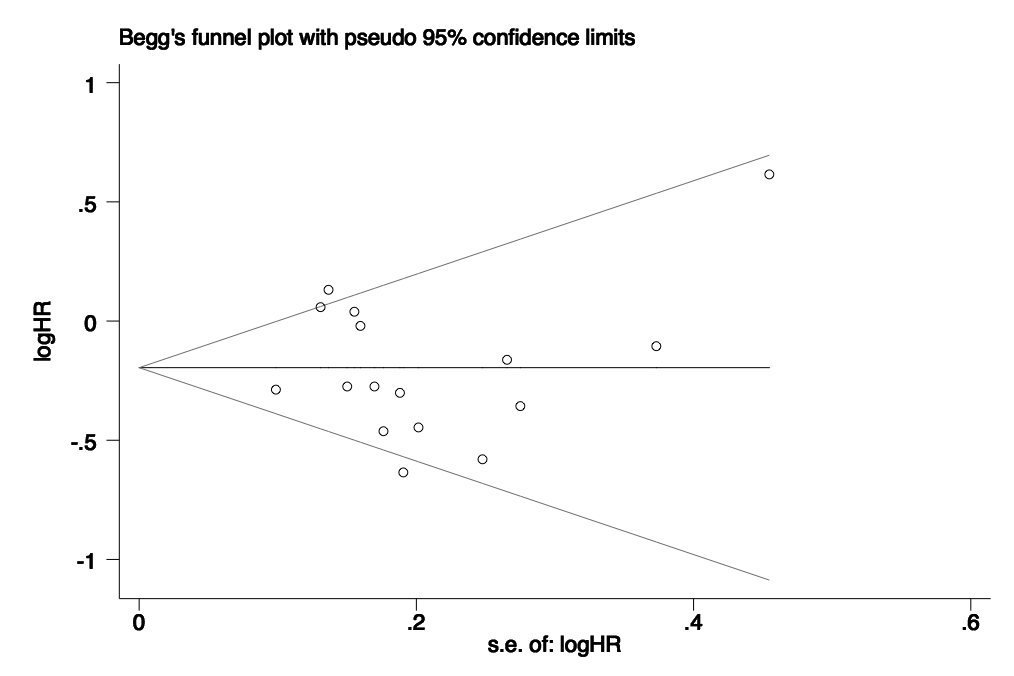


**C**


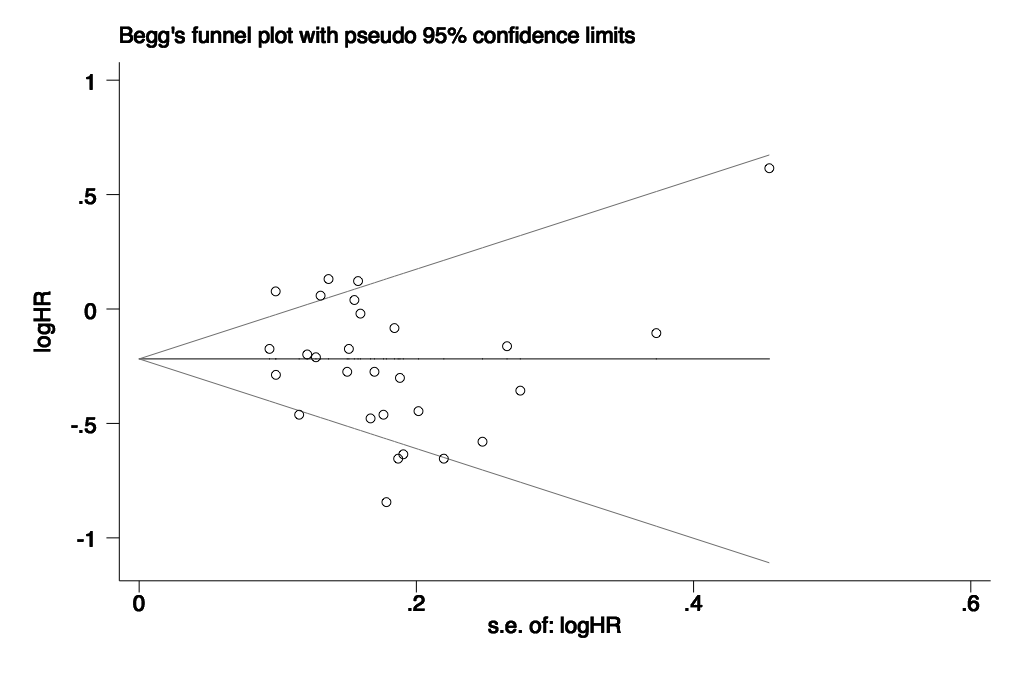


**Supplementary** **Figure 15** - Sensitivity analysis from younger, older and both arms: Sensitivity analysis of overall survival from younger (A), older (B) and both arms (C) in included RCTs for the robustness of findings to different aspects of the trials methodology.

**A**


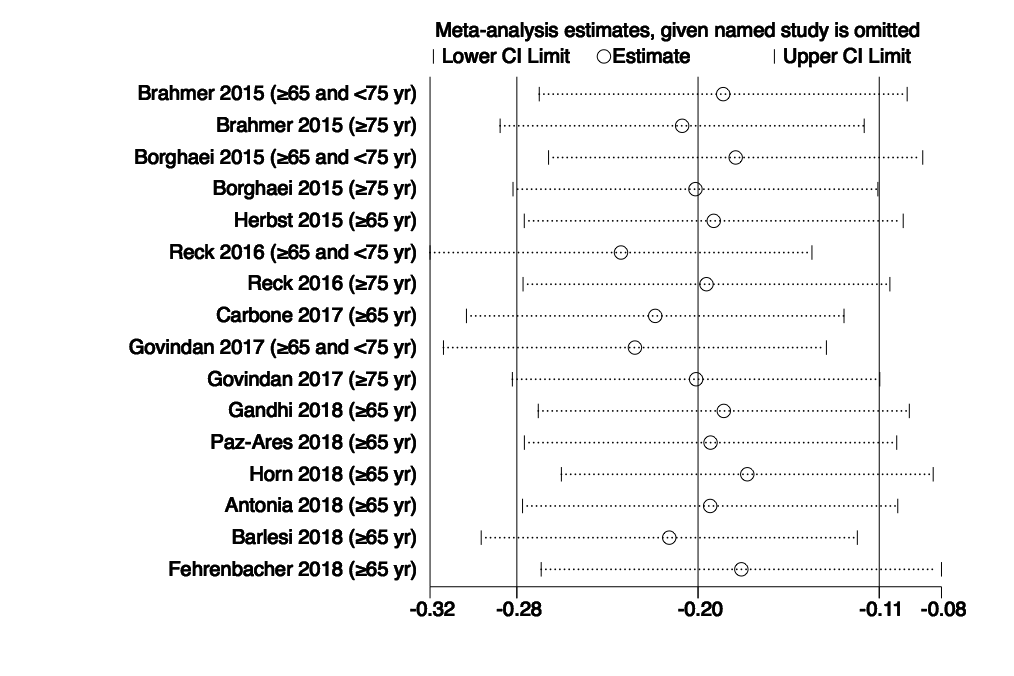
**
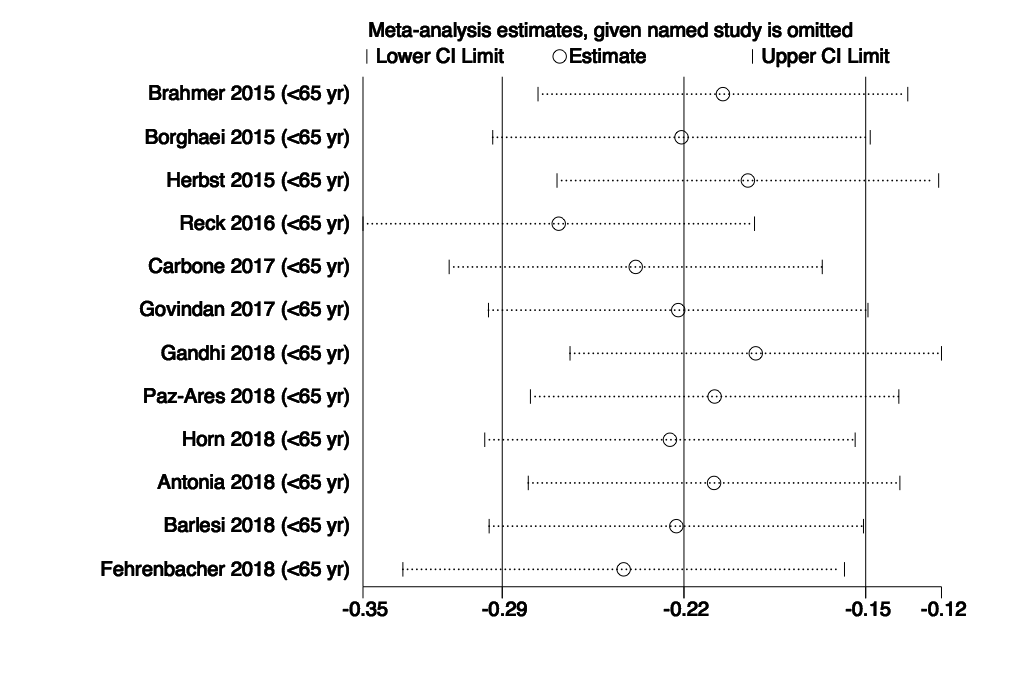
B
C**


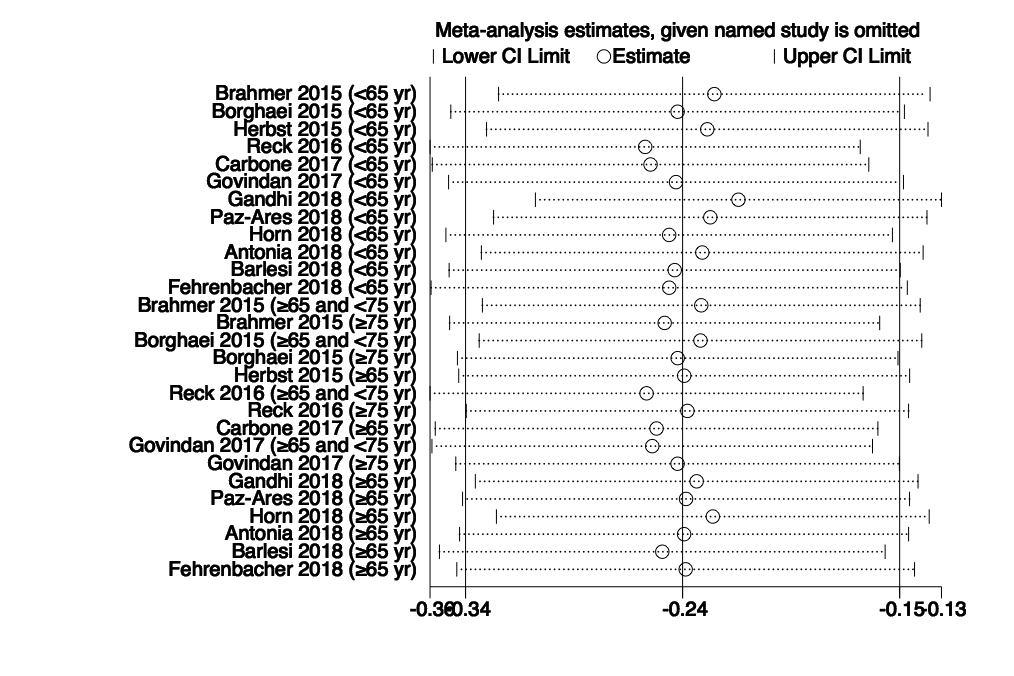

Supplement: Supplementary Materials — Supplementary Tables Supplementary Table 1 -Further Characteristics of the included trial Supplementary Figures Supplementary Figure 1 – Risk of bias graph Supplementary Figure 2 – Risk of bias summary Supplementary Figure 3 – Survival According to a further cut-off Supplementary Figure 4 – Subgroup analysis of class of ICIs Supplementary Figure 5 – Subgroup analysis of histotype Supplementary Figure 6 – Subgroup analysis of line of treatment Supplementary Figure 7 – Subgroup analysis of masking method Supplementary Figure 8 – Subgroup analysis of type of ICIs Supplementary Figure 9 – Subgroup analysis of pathology Supplementary Figure 10 – Meta-Regression Analysis from younger patients Supplementary Figure 11 – Meta-Regression Analysis from older patients Supplementary Figure 12 – Funnel plot from younger and older arms Supplementary Figure 13 – Egger's test from younger, older and both arms Supplementary Figure 14 – Begg's test from younger, older and both arms Supplementary Figure 15 – Sensitivity analysis from younger, older and both arms. [file 9853701.f1.docx]
